# Classification of pulmonary inflammation stages and assessment of multicomponent drug intervention based on spatiotemporal imaging variations utilizing RhB-conjugated poly-L-lysine nanoparticles

**DOI:** 10.1016/j.jpha.2026.101582

**Published:** 2026-02-09

**Authors:** Man Zhang, Shanshan Zhai, He Gao, Tong Sun, Kaixin Liu, Wenshuang Wang, Yuanyuan Hou, Gang Bai

**Affiliations:** State Key Laboratory of Medicinal Chemical Biology, College of Pharmacy and Tianjin Key Laboratory of Molecular Drug Research, Nankai University, Tianjin, 300353, China

**Keywords:** Rhodamine B-conjugated poly-L-lysine, Multimodal probe, Pulmonary inflammation, Disease staging, Multicomponent drug intervention

## Abstract

Acute pulmonary inflammation, a major cause of morbidity and mortality, involves complex immune responses and increased vascular permeability. Conventional imaging techniques for assessing pneumonia and monitoring vascular leakage have limitations in spatial resolution and practicality. Rhodamine B-conjugated poly-L-lysine (RhB-PLL) self-assembled nanoparticles (NPs) were synthesized for targeting mitochondria and sensing vascular permeability. Dynamic spatiotemporal imaging analysis stratifies the progression of acute pneumonia into four distinct stages. Six phosphatidylcholine (PC) metabolites, exhibiting stage-dependent variations, were proposed as potential biomarkers to support RhB-PLL detection. Subsequently, Fuzheng Jiedu decoction (FZJD) was used as a model to validate the rationale of syndrome-specific treatment for pneumonia. Three simplified prescriptions derived from FZJD showed unique advantages at different stages. Xiaochaihu decoction (XCH) relieved inflammation and oxidative stress in mild to moderate cases; Sanren decoction (SR) reduced vascular leakage and prevented pulmonary edema in moderate to severe cases; Maxingshigan decoction (MXSG) improved mitochondrial function and prevented microcirculatory disorders in severe to critical cases. The combined prescription, FZJD, mitigated disease progression in mild to moderate cases and demonstrated the benefits of overall intervention. This conclusion was further substantiated by metabolomics cluster analysis. Real-time RhB-PLL multimodal imaging enables precise visualization and detection of inflammation progress, facilitates therapy assessment, and enhances the understanding of multicomponent drug combinations.

## Highlights

• RhB-PLL nanoparticles enable real-time dual imaging of mitochondrial dysfunction and vascular permeability in pneumonia.
• RhB-PLL imaging stratifies acute pneumonia progression into four distinct stages.
• RhB-PLL nanoparticles enable TCM multicomponent drug intervention assessment in pneumonia.

## Graphical abstract

**Figure:**
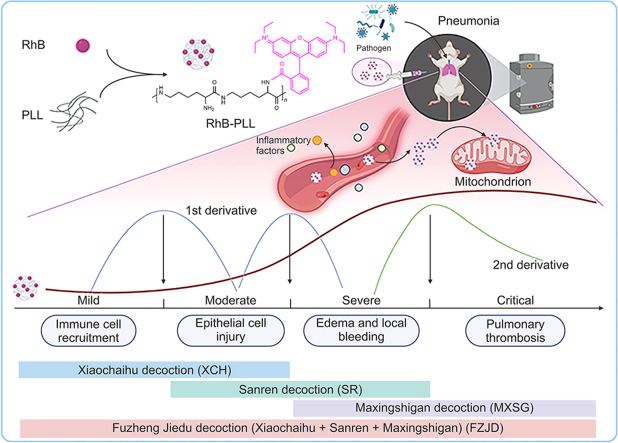

## 1 Introduction

Acute pulmonary inflammation, a major cause of morbidity and mortality in infectious diseases, presents significant challenges in clinical management [1]. When pathogens invade, respiratory epithelial cells detect pathogen-associated molecular patterns (PAMPs) via pattern recognition receptors (PRRs), triggering the release of interferons and pro-inflammatory mediators [2]. These molecules recruit and activate immune cells. Lung epithelial cells and macrophages release chemokines that promote apoptosis, increase capillary permeability, and attract neutrophils and monocytes [3]. Neutrophil infiltration into the alveolar space and interstitium damages epithelial and endothelial cells, disrupting the alveolar-capillary barrier and causing edema due to imbalanced protein transport [4]. The coagulation cascade then activates, leading to capillary blockage, congestion, and thrombosis [5]. This process involves immune activation, tissue injury, and coagulation. Monitoring this complex progression *in vivo* remains a significant challenge.

The dynamic changes in vascular permeability during the progression of pneumonia serve as a critical indicator for evaluating both the intensity and stage of the inflammatory response, making it an invaluable parameter for disease staging [6]. Currently, the main methods for detecting pulmonary vascular permeability are optical and non-optical imaging techniques. Optical imaging relies mainly on fluorescently labeled dextran or other plasma markers to monitor vascular leakage dynamics in real time using *in vivo* fluorescence imaging or two-photon laser scanning microscopy [7]. While two-photon imaging offers high-resolution observation of individual blood vessels, it has limited applicability and is invasive for experimental animals [8]. Non-optical imaging techniques like positron emission tomography (PET) and magnetic resonance imaging (MRI) use radioactive markers or contrast agents to measure vascular permeability changes for whole-body imaging [9]. PET is clinically used to assess pulmonary alveolar leakage to evaluate the endothelial barrier function of the lungs [10]. For example, vascular permeability can be quantified by measuring protein exudate leakage using radioactive tracers such as ^68^Ga-transferrin and ^11^C-methionine albumin (ALB) [11]. Gadolinium-diethylene triamine pentaacetic acid (Gd-DTPA) and bovine serum ALB Gd-DTPA (BSA Gd-DTPA) are frequently employed as contrast agents in MRI to observe permeability alterations and quantify inflammatory exudation [12,13]. However, these methods are limited by low spatial and temporal resolution, radiation exposure, and high complexity and cost of equipment.

A staging-based diagnostic and therapeutic strategy is essential for accurate disease prognosis. By mitigating excessive immune responses in the early stages, potential lung tissue damage can be substantially reduced. In the mid-to-late stages, addressing endothelial injury and reducing edema are critical for preventing further deterioration [14]. This phase-specific treatment approach alleviates parenchymal damage, reduces the risk of systemic complications, and ultimately enhances recovery rates in patients with pulmonary inflammation. Clinicians currently lack an objective, dynamic, and quantitative tool that can precisely delineate the transition from mild inflammation to severe microvascular leakage, mitochondrial dysfunction, and thrombosis. This critical gap hampers timely intervention and personalized therapy. Therefore, there is an urgent need to develop a convenient and feasible method for pneumonia staging to enable precise treatment.

For an extended period, traditional Chinese medicine (TCM) has lacked quantitative spatiotemporal imaging methods for effectively monitoring treatment efficacy *in vivo*. In this study, rhodamine B-conjugated poly-L-lysine (RhB-PLL) multimodal nanoparticles (NPs) were designed based on enhanced vascular permeability and mitochondrial membrane depolarization. RhB-PLL imaging allows real-time visualization of vascular leakage and continuous monitoring of lung function during pulmonary infection. This multidimensional and continuous diagnostic approach provides a valuable tool for assessing disease progression and pulmonary inflammation at all stages. To evaluate the utility of this imaging system, we applied it to investigate the therapeutic efficacy of Fuzheng Jiedu decoction (FZJD), a clinically validated TCM formula widely used for pneumonia. Furthermore, three simplified prescriptions derived from FZJD were analyzed to elucidate their respective therapeutic advantages at specific stages of disease progression. This approach not only demonstrates the stage-dependent effects of individual formulas but also underscores the potential mechanisms of TCM as a holistic intervention strategy in pneumonia.

## 2 Methods and materials

### 2.1 Materials and reagents

All chemical reagents were of analytical grade and used without further purification. *N*-hydroxysuccinimide (NHS) was sourced from Alfa Aesar (Shanghai, China). 1-Ethyl-3-(3-dimethylaminopropyl) carbodiimide hydrochloride (EDC HCl) was procured from Huawai Ruike Chemical (Beijing, China). Triethylamine was obtained from Inoke Technology (Beijing, China). RhB and avibactam sodium were purchased from Aladdin Bio-Chem Technology (Shanghai, China). PLL was supplied by Bainaifu Bioengineering (Zhengzhou, China). Ethylenediamine tetraacetic acid (EDTA) was obtained from Guangfu Technology (Tianjin, China). Column Chromatography Silica Gel (200–300 mesh) was acquired from Bohua Chemical Reagent (Tianjin, China). Ceftazidime was obtained from Solaibao Technology (Beijing, China). Mitochondrial import receptor subunit TOM20 homolog (TOM20) recombinant antibody (80501-1-RR) were purchased from Proteintech Wuhan Sanying (Wuhan, China). Primary antibodies against anti-mouse myeloperoxidase (MPO) (ab300650) and anti-histone H3 (citrulline R2+R8+R17) (ab5103) were purchased from Abcam (Cambridge, UK). Goat anti-rabbit IgG H&L (Alexa Fluor® 488, ab150077) were purchased from Abcam and rhodamine tetramethylrhodamine isothiocyanate (TRITC))-conjugated goat anti-rat IgG (H+L) (SA00007-7) were purchased from Proteintech Wuhan Sanying. 4′,6-Diamidino-2′-phenylindole (DAPI) (C0065) were purchased from Beijing Solarbio Science & Technology Co., Ltd. (Beijing, China). *Pseudomonas aeruginosa* 14 (*PA 14*) strain was obtained from Professor Bai Fang group of Nankai University (Tianjin, China). *PA* antibody (1001/214) (Alexa Fluor® 488) was obtained from Novus Biologicals‌ (Beijing, China). The hematoxylin and eosin (H&E) staining kit (G1120) was purchased from Solaibao Technology. The modified Masson's trichrome (MSB) staining kit (DG0075) was purchased from Beijing Leagene Biotechnology Co., Ltd. (Beijing, China). Adenosine triphosphate/adenosine diphosphate (ATP/ADP) Ratio Chemiluminescence Assay kit was purchased from Elabscience Biotechnology Co., Ltd. (Wuhan, China). All traditional Chinese medicinal materials in FZJD, Xiaochaihu decoction (XCH), Sanren decoction (SR), and Maxingshigan decoction (MXSG) were procured from Beijing Tong Ren Tang Chinese Medicine Co., Ltd. (Beijing, China) and identified as the medicinal herbs by the Department of Medicinal Botany, Nankai University. The compositional and preparation details of these prescriptions, together with their active ingredient standards and the liquid chromatography (LC) identification results for each decoction, were provided in the Supplementary data and Figs. S1–S4.

### 2.2 Synthesis of RhB-PLL NPs

958.1 mg (2 mmol, 1 equivalent) RhB was dissolved in 15 mL of dichloromethane, followed by the addition of 253.2 mg (2.2 mmol, 1.1 equivalent) of NHS and 383.4 mg (2 mmol, 1 equivalent) of EDC HCl to react overnight at room temperature. The supernatant, concentrated under vacuum, was purified by silica gel column chromatography with dichloromethane:methanol (25:1, *V*/*V*). The yield of NHS-RhB was around 51.3%. 200 mg (0.05 mmol, 1 equivalent) of PLL was dissolved in 0.5 mL of deionized water, to which 30 μL (0.2 mmol) of triethylamine was added to form a solution. Concurrently, 135 mg (0.25 mmol, 5 equivalents) of NHS-RhB was dissolved in 1 mL of acetonitrile at ambient temperature. The PLL solution was added dropwise to the NHS-RhB solution under continuous stirring at room temperature for 24 h, forming self-assembly NPs. The mixture was transferred to a pre-equilibrated dialysis bag (3500 Da, 22 mm) and dialyzed against deionized water for three days. The resulting purple-red solution was lyophilized, yielding 94.8 mg of purple-red fluorescent RhB-PLL NPs. The overall yield was approximately 30.1%. The structures of RhB-PLL were characterized by Fourier transform infrared spectroscopy (FT-IR), nuclear magnetic resonance (NMR) spectroscopy, and high-resolution mass spectrometry (HRMS).

### 2.3 Characterization of RhB-PLL NPs

RhB-PLL was coated with platinum using a Quorum SC7620 Vacuum Coater (Quorum, Lewes, UK) at an amperage of 10 mA, with a spraying time of 45 s. Scanning electron microscopy (SEM) (TESCAN MIRA LMS; TESCAN, Brno, Czech Republic) was performed to acquire electronic micrographs at an operating voltage of 3 kV. The physicochemical properties of RhB-PLL, including hydrodynamic diameter, polydispersity index (PDI), and zeta potential, were assessed using a dynamic light scattering (DLS) instrument (Malvern Zetasizer NanoZS, Malvern, UK) at 25 °C [15]. Prior to measurement, RhB-PLL was dissolved in deionized water at a concentration of 0.5 mg/mL and subsequently filtered through a 0.22-μm filter. RhB-PLL was then reconstituted in phosphate-buffered saline (PBS), and changes in particle size and PDI of micelles were measured for 48 h.

To understand its optical properties, RhB-PLL was dissolved in ultrapure water to a concentration of 0.1 mg/mL for spectral characterization. Ultraviolet-visible spectroscopy (UV-vis) spectra were recorded using a spectrophotometer after baseline calibration with ultrapure water, with scans performed from 200 to 800 nm. Fluorescence spectra were obtained using a microplate reader (Tecan Spark, Tecan, Grödig, Austria). The excitation spectrum was scanned at an emission wavelength of 600 nm to identify the excitation peak, followed by scanning the emission spectrum based on this excitation wavelength.

### 2.4 Animal welfare and protocols

Male Kunming mice (18–22 g) were purchased from Beijing Vital River Laboratory Animal Technology (Beijing, China). The animals were randomly assigned to cages with free access to water and standard mouse chow at 25 °C and were allowed to acclimate for one week before the experiment. All animal experiments were carried out in accordance with the National Institute of Health (NIH) Guide for the Care and Use of Laboratory Animals and were approved by the Nankai University of Laboratory Animals Care and Use Committee of Nankai University (Approval No.: 2022-SYDWLL-000491). In subsequent animal experiments, five animals per group were used for nontargeted metabolomics analysis, and six animals per group were used for other experiments. Full details of group names, treatment protocols, and doses are provided in Table S1.

### 2.5 Acute pulmonary inflammation

An acute pulmonary inflammation mouse model was established by intranasal instillation of *PA 14* strain (4 × 10^7^ CFU/20 μL in PBS) into anesthetized mice [16]. 7 h post-infection, mice were randomly assigned to the following groups: control (i.p. saline), model (*PA 14* infection), CAT (i.p. ceftazidime 400 mg/kg and avibactam 100 mg/kg), FZJD (oral FZJD decoction, 22 g/kg), XCH (oral XCH decoction, 9 g/kg), SR (oral SR decoction, 7 g/kg), and MXSG (oral MXSG decoction, 7 g/kg). After infection and drug intervention, the mice were sacrificed at designated time points, and lung tissues were sectioned for histological staining including H&E and modified MSB, followed by subsequent analysis. Immunofluorescence staining using an anti-*PA* antibody was performed to detect *PA 14* strain infection in lung tissue sections.

### 2.6 In vivo imaging assay of RhB-PLL

Mice were intravenously injected with 25 mg/kg RhB-PLL via the tail vein at specified time points. Fluorescence was continuously monitored using an *in vivo* small animal imaging system (Ex = 530 nm, Em = 600 nm, exposure time 1 s) (NightOWL II LB983, Berthold Technologies, Bad Wildbad, Germany) to assess therapeutic effects. The acquired images were processed using Indigo image processing software. The average fluorescence intensity in the lung region was calculated, and fluorescence accumulation was quantitatively evaluated.

### 2.7 Immunofluorescence assay

Frozen lung tissue sections were washed with PBS and subsequently fixed with 4% paraformaldehyde at room temperature for 30 min. After fixation, the sections were blocked with 5% goat serum for 1 h to minimize nonspecific binding. Next, sections were incubated overnight at 4 °C with the primary antibodies, including anti-TOM20, anti-MPO, anti-histone H3, and anti-*PA* antibody. Following overnight incubation, sections were incubated for next 1 h at 37 °C in the dark with secondary antibodies, either rhodamine (TRITC)-conjugated Goat anti-Rat IgG (H+L) or Alexa Fluor® 488-conjugated goat anti-rabbit IgG (H+L). After thorough washing with PBS, the sections were counterstained with DAPI, and the fluorescence signals were analyzed using confocal microscopy (TCS SP8, Leica, Mannheim, Germany).

### 2.8 Reactive oxygen species (ROS) level detected by *Cu-luminol* (Cu-Lum)@NPs

A ROS-responsive luminescent Cu-Lum@NPs probe was employed to detect ROS levels [16]. In brief, 100 μL of freshly prepared mouse lung tissue lysate was added to a 96-well plate, followed by the addition of 5 μg/mL Cu-Lum@NPs. Subsequently, the luminescence intensity of the samples was measured using a multifunctional microplate reader.

### 2.9 Nontargeted metabolomics

Blood samples were collected from mice via the retro-orbital plexus for serum extraction. Following a 30 min clotting period at room temperature, the samples were centrifuged at 3000 *g* for 10 min at 4 °C. Non-targeted metabolomics analysis was performed by Wuhan Maiwei Metabolomics Biotechnology Co., Ltd. (Wuhan, China). Serum samples were extracted with methanol:water (4:1, *V*/*V*) and analyzed by LC-mass spectrometry (MS) in positive and negative ion modes. Chromatographic separation was performed on a C_18_ column (Nacalai Tesque, Kyoto, Japan) with gradient elution. The mass spectrometer was operated in full scan mode (*m*/*z* 50–1000). Data were processed with various forms (X) of chromatography mass spectrometry (XCMS) and MetaboAnalyst for peak detection, alignment, and statistical analysis. Orthogonal partial least squares discriminant analysis (OPLS-DA) was conducted to explore metabolic differences among groups. Kyoto Encyclopedia of Genes and Genomes (KEGG) pathway enrichment was analyzed based on differential metabolites, with *P*-values from hypergeometric tests.

### 2.10 ATP/ADP ratio detection

Fresh lung tissues were ground into single-cell suspensions and examined according to the manufacturer's instructions.

### 2.11 Statistical analysis

Data are expressed as mean ± standard deviation (SD). Statistical analyses were performed using GraphPad Prism 10. Significant differences between two groups were analyzed by Student's *t*-test; differences among multiple groups were analyzed by one-way analysis of variance (ANOVA).

## 3 Results

### 3.1 Synthesis and characterization of RhB-PLL

**Fig. 1:**
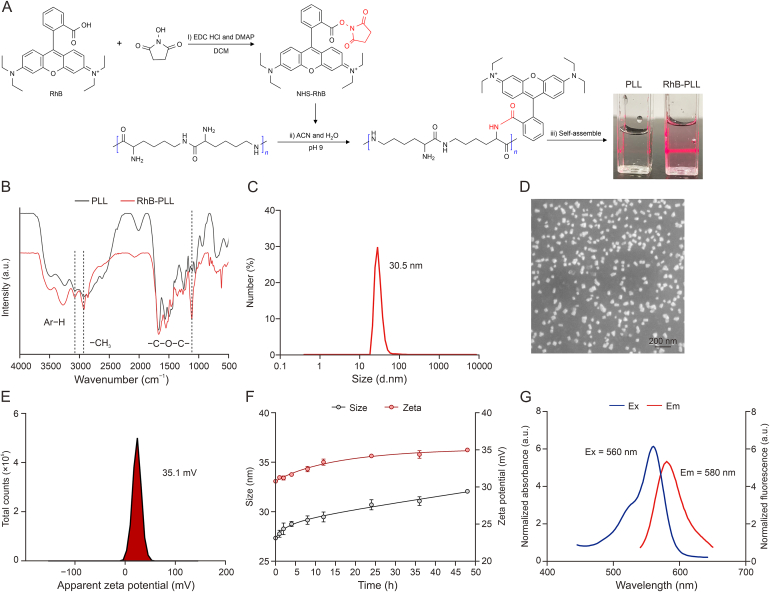
Preparation and characterization of the rhodamine B-conjugated poly-L-lysine (RhB-PLL) nanoparticles (NPs). (A) The images indicate the synthesis route of RhB-PLL. (B) Structural comparison of Fourier transform infrared spectroscopy (FT-IR) spectra between RhB-PLL and PLL. (C) Particle size distribution of RhB-PLL measured by dynamic light scattering (DLS). (D) Scanning electron microscopy (SEM) images of RhB-PLL. (E) Zeta potential of RhB-PLL. (F) Stability variation of RhB-PLL over 48 h in size and zeta potential. Data presented as mean ± standard deviation (SD) (*n* = 3). (G) Fluorescence excitation and emission spectrum scanning of RhB-PLL. EDC HCl: 1-ethyl-3-(3-dimethylaminopropyl) carbodiimide hydrochloride; DMAP: 4-dimethylaminopyridine; DCM: dichloromethane; NHS: *N*-hydroxysuccinimide; ACN: acetonitrile.

Amino acid-based NPs are extensively utilized in biomedical applications owing to their biocompatibility, degradability, and functional versatility. For example, histidine-coated cerium oxide nanocages acts as a cell-penetrating peptide, enhancing corneal permeability and facilitating pH-responsive controlled release of acetylcholine and SB431542, which significantly improves therapeutic outcomes for chemical corneal injuries [17]. PLL polymer materials have also been widely utilized for site-specific drug delivery due to their excellent biocompatibility, positive surface charge, and ability to enhance cell adhesion [18]. RhB, with a partial positive charge for targeting mitochondria, offers good tissue penetrating fluorescence, efficient conjugation to PLL, and cost-effectiveness over other dyes [19]. Additionally, studies have demonstrated that cationic NPs (20–200 nm) are retained within lung cells for extended periods [20]. Building on these advancements, RhB-conjugated PLL self-assembled NPs were designed and synthesized for the detection of pulmonary vascular permeability and mitochondrial targeting in this study. The synthesis of RhB-PLL was carried out according to the synthetic route (Fig. 1A). Compared to unlabeled PLL, the RhB-PLL exhibited distinct colloidal characteristics with the Tyndall effect. The FT-IR spectrum of RhB-PLL exhibited significant increases in the –C–O–C group absorption between 1000 and 1250 cm^−1^, the characteristic peak of the benzene ring –H at 3000–3300 cm^−1^, and the –CH_3_ characteristic peak at 2800–3000 cm^−1^ when compared to ε-PLL (Fig. 1B). The structural integrity of RhB-PLL was further confirmed using NMR and HMRS (Figs. S5–S9). Quantitative analysis of the feed and residual raw materials revealed that each ε-PLL polymer bound approximately three RhB molecules (Fig. S10).

The DLS analysis showed that the average particle size of RhB-PLL was about 30.5 nm, with a size distribution ranging from 15 to 40 nm (Fig. 1C), which falls within the optimal size range reported to enhance mucus penetration, promote alveolar epithelial cell uptake, and enable efficient delivery to deep lung tissues [21]. SEM images indicated RhB-PLL exhibited a uniform shape with good dispersion (Fig. 1D). The zeta potential was about 35.1 mV (Fig. 1E), and it markedly decreased to +2.99 mV following EDTA treatment (Fig. S11). To assess the colloidal stability of RhB-PLL, its average particle size and zeta potential were monitored in both PBS and serum-containing medium over a 48-h period. In PBS, RhB-PLL remained relatively stable, with particle size increasing modestly from 30.5 to 32 nm. In contrast, in 10% serum-containing PBS, the average particle size increased from 28.5 nm to approximately 60 nm, likely due to the adsorption of serum proteins forming a protein corona. The zeta potential also decreased from its initial value but remained stably positive, around +19 mV up to 48 h, which is considered favorable for maintaining mitochondrial targeting capability and colloidal stability (Figs. 1F and S12). Furthermore, the spectral scanning analysis revealed that the optimal excitation wavelength for RhB-PLL was 560 nm, and the corresponding emission wavelength was 580 nm (Fig. 1G).

### 3.2 The imaging distribution preference of RhB-PLL NPs

**Fig. 2:**
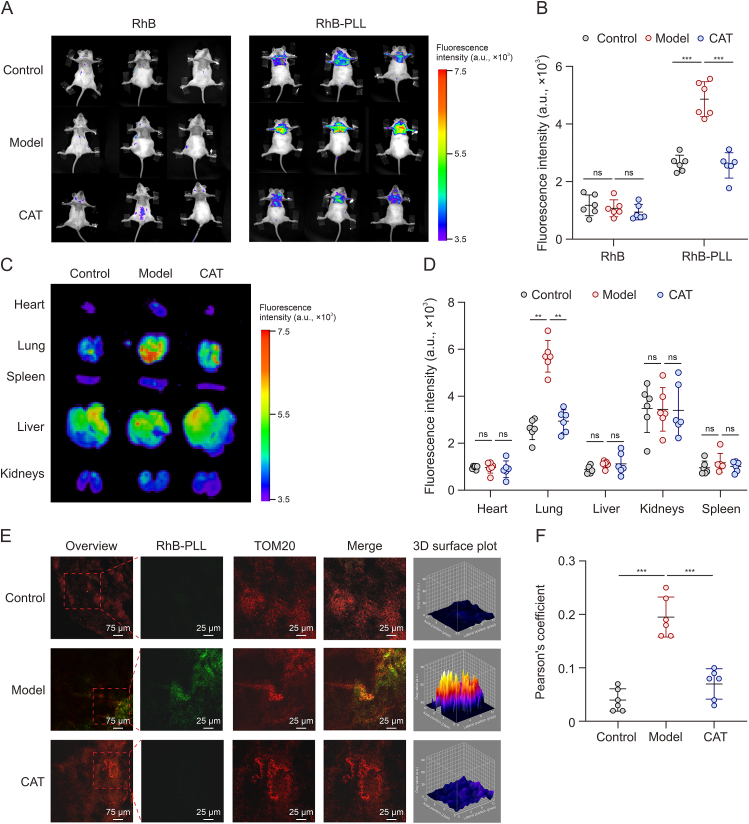
*In vivo* investigation of the fluorescence imaging distribution and biodistribution of rhodamine B-conjugated poly-L-lysine (RhB-PLL) nanoparticles (NPs) in a pneumonia mouse model. (A, B) *In vivo* imaging of RhB-PLL (A) and statistics in lung tissue (B). (C, D) Organ distribution of RhB-PLL (C) and statistical evaluation (D). (E, F) Co-localization analysis of RhB-PLL and mitochondrial marker mitochondrial import receptor subunit TOM20 homolog (TOM20) in lung sections (E) and Pearson's coefficient statistics (F) (RhB-PLL, pseudo green; TOM20, pseudo red). Data presented as mean ± standard deviation (SD) (*n* = 6). ^∗∗^*P* < 0.01 and ^∗∗∗^*P* < 0.01. ns: not significant. CAT: i.p. ceftazidime 400 mg/kg and avibactam 100 mg/kg; 3D: three-dimensional.

Intact alveolar epithelial and endothelial cells form tight junctions that regulate fluid and inflammatory cell movement into the interstitial space. Acute pulmonary inflammation increases microvascular permeability, leading to inflammatory cell infiltration and pulmonary edema. To test RhB-PLL probe feasibility for *in vivo* detection, the *PA 14* strain was administered to mice to induce acute pulmonary inflammation. After modeling, the 24-h survival rate decreased to 20% in the model group, whereas treatment with the antibiotic CAT increased the survival rate to 80% (Fig. S13). *In vivo* imaging experiments demonstrated that, compared to free RhB molecules, RhB-PLL NPs exhibited significantly enhanced accumulation in the lungs. Moreover, the model group showed significantly higher fluorescence intensity than the control group at 12 h post-administration. After CAT treatment, the fluorescence intensity was markedly diminished, indicating a potential reduction in vascular leakage (Figs. 2A and B). Evans blue staining results were consistent with the *in vivo* imaging experiments (Figs. S14 and S15). Subsequently, the organs were carefully excised from the mice for RhB-PLL distribution imaging analysis. Although RhB-PLL NPs exhibited effective liver targeting, increased vascular permeability led to notable fluorescence variations in lung tissue across groups at 16 h (Figs. 2C and D). Compared with the control and CAT-treated groups, the model group exhibited significantly increased accumulation of RhB-PLL in both pulmonary macrophages (CD68-positive) and epithelial cells (E-cadherin-positive) (Fig. S16). Biodistribution analysis in healthy mice for 24 h further revealed weak fluorescence in the heart, kidneys, and spleen, while the liver consistently displayed strong fluorescence (Fig. S17). In contrast, the enhanced pulmonary fluorescence and intergroup differences observed in the lung injury model disappeared, indicating that RhB-PLL selectively accumulates at sites of pulmonary inflammation rather than in normal lung tissue.

Given the characterization of mitochondrial membrane potential (ΔΨm), the mitochondrial targeting of RhB-PLL was examined using immunofluorescence assays on pulmonary tissue sections. In the model group, RhB-PLL demonstrated superior co-localization with TOM20, a marker protein of the mitochondrial outer membrane, compared to the control group (Figs. 2E and F). This effect was attenuated in the CAT treatment group, suggesting that RhB-PLL mitochondrial accumulation was enhanced under pneumonia conditions. This phenomenon may be attributed to ROS overproduction in inflammatory microenvironments, which disrupts mitochondrial homeostasis [22]. The increased exposure of negative charges on the inner membrane of depolarized mitochondria facilitates the accumulation of the positively charged complex via electrostatic interactions [23]. To further evaluate the mitochondrial targeting capability of RhB-PLL, an LPS-induced inflammatory model was established in RAW264.7 cells. The results demonstrated that RhB-PLL exhibited significantly enhanced mitochondrial targeting in the model group compared to both the control group and the CAT-treated group. In contrast, RhB alone showed no mitochondrial targeting across all groups. Notably, when EDTA was introduced to neutralize the negative charge of RhB-PLL, its mitochondrial targeting capacity was abolished, further confirming that the mitochondrial accumulation of RhB-PLL is dependent on its negative surface charge (Fig. S18).

### 3.3 Classification of pneumonia stages based on RhB-PLL spatiotemporal imaging

**Fig. 3:**
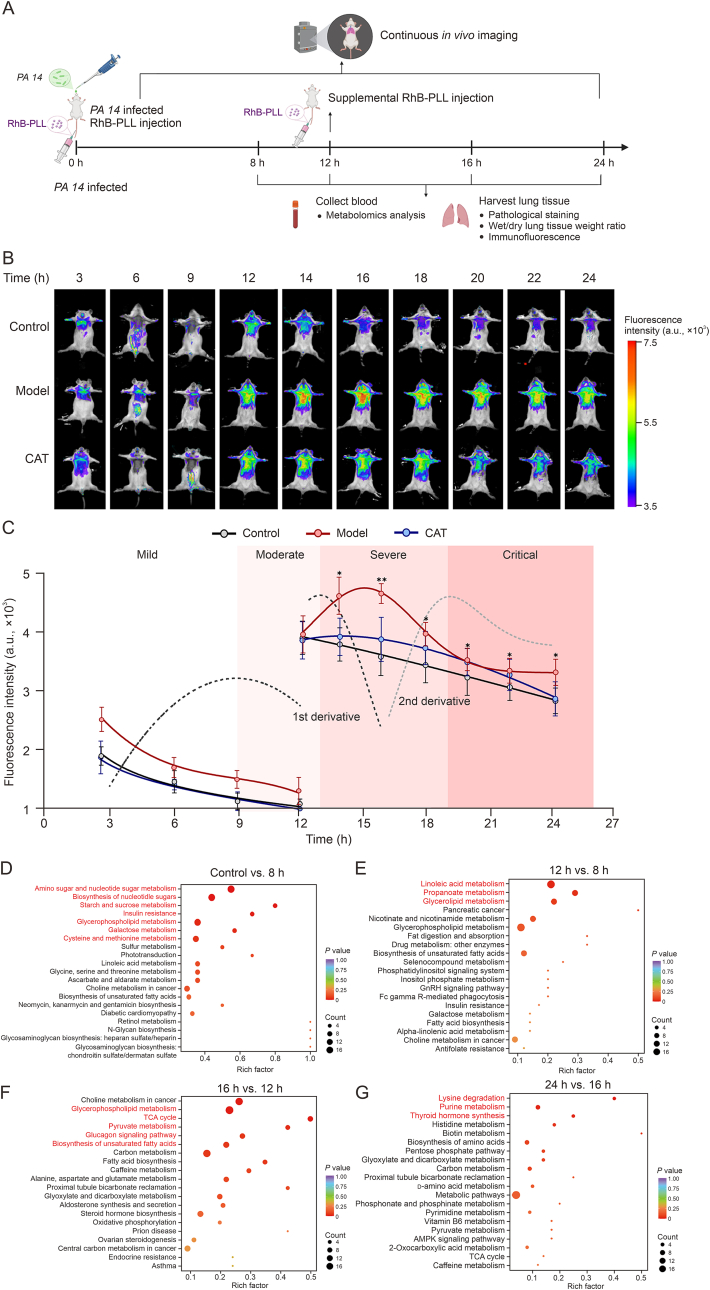
Rhodamine B-conjugated poly-L-lysine (RhB-PLL) spatiotemporal imaging and metabolomics analysis for assessing disease progression and staging. (A) Diagram of the experimental procedure and administration protocol in a pneumonia mouse model. (B) *In vivo* RhB-PLL imaging detection within a 24-h period. (C) Fitting curve of the average fluorescence intensity for the first or second derivative (*n* = 6). Data presented as mean ± standard deviation (SD). ^∗^*P* < 0.05 and ^∗∗^*P* < 0.01 vs. control group. (D–G) Enrichment analysis of Kyoto Encyclopedia of Genes and Genomes (KEGG) differential pathways in two adjacent interval points: control vs. 8 h (D), 12 h vs. 8 h (E), 16 h vs. 12 h (F), and 24 h vs. 16 h (G). *P*-value was the hypergeometric test *P*-value. Pathways with ^∗^*P* < 0.05 was marked in red (*n* = 5). *PA 14*: *Pseudomonas aeruginosa* 14; CAT: i.p. ceftazidime 400 mg/kg and avibactam 100 mg/kg; GnRH: gonadotropin-releasing hormone; TCA: tricarboxylic acid; AMPK: adenosine monophosphate (AMP)-activated protein kinase.

To enable continuous monitoring of pneumonia progression, RhB-PLL was administered at both 0 and 12 h post-infection to maintain adequate fluorescence signal throughout the 24 h observation period. Subsequently, the fluorescence intensity within lung tissues was measured in real-time using an *in vivo* imaging system (Fig. 3A). Continuous 24 h monitoring revealed a significant increase in lung fluorescence intensity in the model group compared to the control group during the period from 12 to 24 h (Fig. 3B). In contrast, the fluorescence intensity in the antibiotic CAT treatment group showed only a slight difference relative to the model group. To identify the trend in the model group from 3 to 24 h, the changes of fluorescence intensity were fitted to first or second derivatives. Based on the inflection point, the infiltration progression was divided into four distinct stages (Fig. 3C): mild stage (0–9 h), moderate stage (9–13 h), severe stage (13–19 h), and critical stage (19–24 h).

To validate the accuracy of RhB-PLL imaging for staging pulmonary inflammation, four time points (8, 12, 16, and 24 h) at each stage were selected for metabolomics and pathological analyses. A total of 2974 metabolites were detected by metabolomics analysis. OPLS-DA showed clear clustering within groups and distinct separation between groups (Fig. S19). Subsequently, differential metabolite pathway enrichment analyses were performed independently for the metabolites that showed significant differences between the two time points (before and after) in the model group. In the mild stage (Fig. 3D), immune cells were activated, glycolysis was enhanced, and changes occurred in several energy metabolic pathways, including amino sugar and nucleotide sugar, starch and sucrose, glycerophospholipid and galactose metabolism, cysteine and methionine metabolism, and insulin resistance. In the moderate stage (Fig. 3E), lipid peroxidation was intensified, with increased metabolism of linoleic acid, propanoate, and glycerolipid, indicating phospholipid degradation within the cell membrane and a further exacerbation of inflammation.

In the severe stage (Fig. 3F), aerobic glycolysis, including the tricarboxylic acid (TCA) cycle and pyruvate metabolism, as well as the glucagon signaling pathway and carbon metabolism, reached their peaks. Meanwhile, the increased metabolism of unsaturated and saturated fatty acids suggested a shift in energy supply towards lipolysis [24]. The heightened metabolic activity of choline and glycerophospholipids indicates cell membrane breakdown, impairing fluid transport and increasing permeability [25]. During the critical phase (Fig. 3G), mitochondrial dysfunction, enhanced protein metabolism, and organ failure were supported by activation of energy metabolism-related pathways, including lysine degradation and purine metabolism [26]. Microcirculatory disorder was further indicated by thyroid hormone signaling and histidine metabolism imbalance [27]. Additionally, cytokine storm, peroxidation reactions, and neutrophil extracellular traps (NETs) were fully activated, resulting in platelet aggregation and thrombosis [28]. These pathway enrichment results support the reliability of RhB-PLL NPs for diagnosis pulmonary inflammation.

### 3.4 Pathological studies support the staging diagnosis using RhB-PLL imaging

**Fig. 4:**
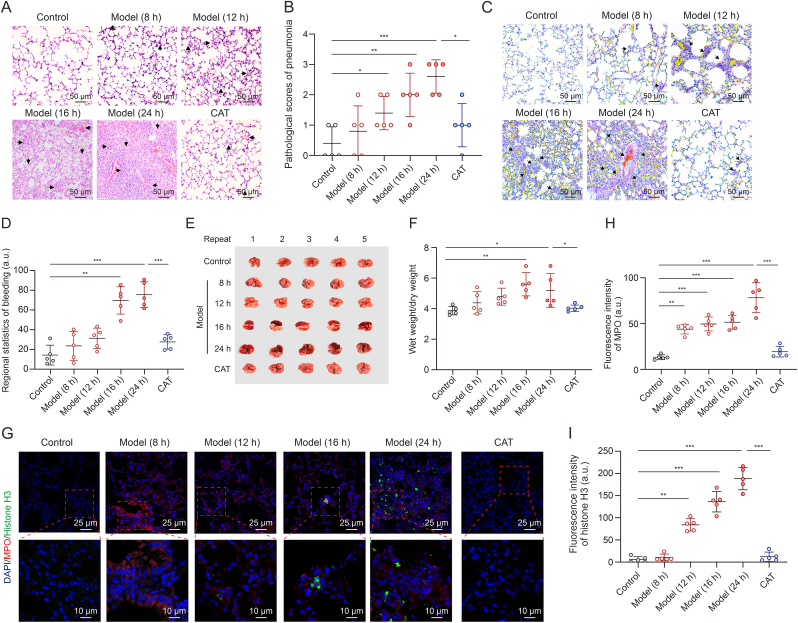
Pathological and immunohistochemical analysis of different stages of pneumonia. (A, B) Hematoxylin and eosin (H&E) staining in the pneumonia course (A) and the corresponding pathological score (B). Pathological parameters in H&E mainly considered hyperemia or hemorrhage in the alveolar wall, intra-alveolar epithelial hyperplasia, emphysema, and inflammatory infiltration. Pathological scores of 0–3 represent normal, mild, moderate, and severe, respectively. (C, D) Modified Masson's trichrome (MSB) staining (C) and the corresponding pathological score for bleeding (D). The red blood cells were stained yellow using Mathew's yellow staining solution, and the area of yellow staining in the image was quantified to determine the pathological score of bleeding. (E, F) Lung tissue morphology changes (E) and wet/dry weight measurements (F). (G) Immunohistochemical image of the nucleus, histone H3, and myeloperoxidase (MPO) markers for neutrophil extracellular traps (NETs) evaluation in the lung tissue section. (H, I) Fluorescence intensity statistics of MPO (H) and hstone-H3 (I) (citrulline R2+R8+R17). Mean fluorescence intensity per tissue section was quantified from five biological replicates for each group. Data presented as mean ± standard deviation (SD) (*n* = 5). The arrows in Figs. 4A and C indicate focal regions of histopathological damage in the lung tissue. ^∗^*P* < 0.05, ^∗∗^*P* < 0.01, and ^∗∗∗^*P* < 0.001. CAT: i.p. ceftazidime 400 mg/kg and avibactam 100 mg/kg; DAPI: 4′,6-diamidino-2′-phenylindole.

To further validate the accuracy of RhB-PLL imaging in staging pneumonia progression, pathological staining was conducted on lung tissues at various stages. As shown in Figs. 4A and B, H&E staining revealed no significant differences at 8 h compared to the control group. By 12 h, mild inflammation and increased interstitial thickness were evident. At 16 h, a notable influx of inflammatory cells and signs of intracellular hemolysis were observed. By 24 h, the inflammatory response had intensified markedly, with evidence of pulmonary capillary hemorrhage. These observations are consistent with the known pathophysiological progression of pneumonia, in which inflammation increases vascular permeability and tissue damage. Additionally, modified MSB staining showed capillary bleeding at 16 h and microvascular embolism at 24 h (Figs. 4C and D), consistent with the interplay between inflammation and microthrombosis in severe respiratory diseases. However, CAT administration markedly attenuated pulmonary inflammation at 24 h.

Similarly, in conjunction with lung morphology and dry-to-wet weight ratio results, significant edema and local bleeding were observed at 16 h (Figs. 4E and F), again reflecting disrupted pulmonary vascular integrity. To assess neutrophil accumulation and NET release, immunofluorescence assays for MPO and histones were performed to further characterize pneumonia progression. Compared with the control group, MPO expression was significantly increased at 8 h, indicating the onset of neutrophil recruitment and activation (Figs. 4G and H). By 12 and 16 h, histone expression exhibited a marked increase, suggesting that neutrophils had transitioned into a hyperactivated state, thereby initiating NETs formation (Fig. 4I), consistent with previously described mechanisms of neutrophil-mediated tissue injury in acute inflammation. At 24 h, a substantial number of NETs were generated, which contributed to the progression of pulmonary thrombosis. These findings further validate the accuracy of RhB-PLL imaging in characterizing pneumonia progression *in vivo*.

### 3.5 Phosphatidylcholines (PCs) are key biomarkers for staging pneumonia progression

**Fig. 5:**
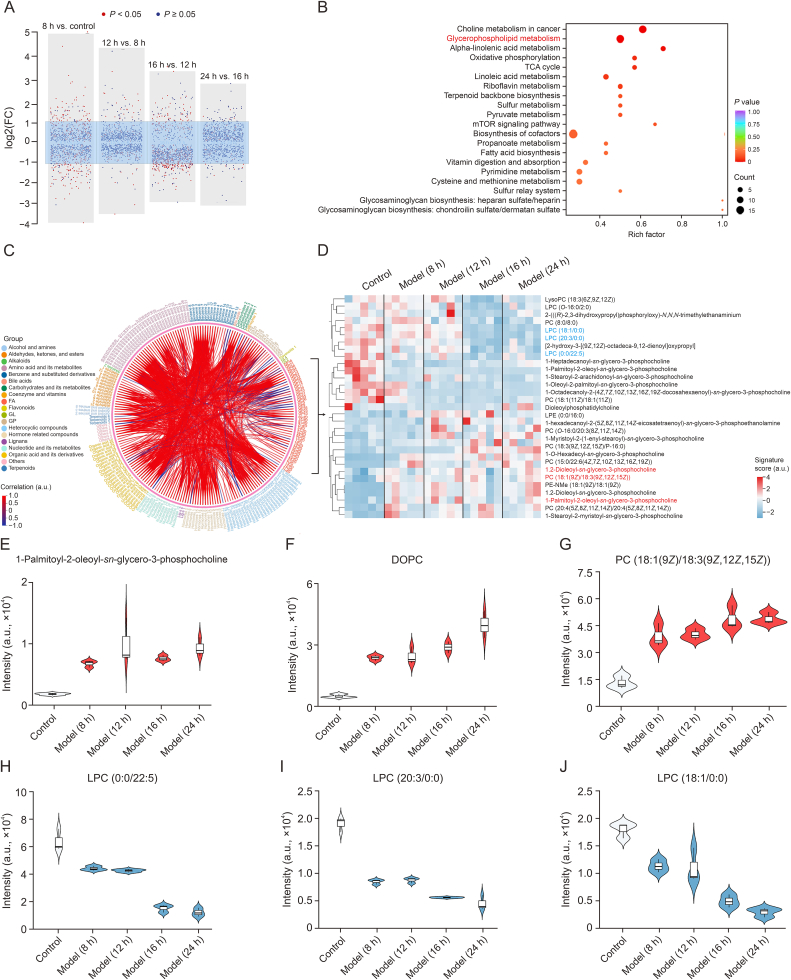
Comprehensive metabolomics analysis identifies potential biomarkers closely linked to pneumonia progression. (A) Volcanic map analysis of differential metabolites across pneumonia progression. (B) Enrichment analysis of Kyoto Encyclopedia of Genes and Genomes (KEGG) pathways at four key time points. (C) Differential metabolite correlation diagram in the pneumonia model. (D) Heatmap analysis of phosphatidylcholine (PC) metabolite clustering at four critical time points. (E–J) Violin map of six representative differential metabolites at four critical time points (*n* = 3): 1-palmitoyl-2-oleoyl-*sn*-glycero-3-phosphocholine (E), 1,2-dioleoyl-*sn*-glycero-3-phosphocholine (DOPC) (F), PC (18:1(9Z)/18:3(9Z,12Z,15Z)) (G), lysophosphatidylcholine (LPC) (0:0/22:5) (H), LPC (20:3/0:0) (I), and LPC (18:1/0:0) (J). FC: fold change; TCA: tricarboxylic acid; mTOR: mammalian target of rapamycin; FA: fatty acyl; GL: glycerolipid; GP: glycerophospholipid; LPE: lysophosphatidylethanolamine; PE: phosphatidylethanolamine.

Identifying of metabolic biomarkers for pulmonary inflammation is essential for early diagnosis, disease staging, treatment guidance, and prognosis evaluation [29]. Therefore, metabolomics data between consecutive time points were reanalyzed (Fig. 5A). As a result, the most significantly enriched pathway associated with the development of acute pulmonary inflammation was choline metabolism and glycerophospholipid metabolism (Fig. 5B). Additionally, the most differentially abundant metabolites were associated with PC (Fig. 5C). PC is well established to play a critical role in pulmonary surfactant synthesis and lung function maintenance. However, its dysregulation may contribute to severe conditions such as respiratory failure and increased susceptibility to infections [30]. Lysophosphatidylcholine (LPC), a proinflammatory lipid, facilitates immune cell migration, cytokine production, oxidative stress induction, and apoptosis [31]. LPC can be reconverted to PC by LPC acyltransferase [32]. As shown in Fig. 5D, the heatmap revealed distinct trends in PC and LPC levels during pulmonary inflammation progression. Notably, 1-palmitoyl-2-oleoyl-*sn*-glycero-3-phosphocholine, 1,2-dioleoyl-*sn*-glycero-3-phosphocholine (DOPC), PC (18:1(9Z)/18:3(9Z,12Z,15Z)), LPC (0:0/22:5), LPC (20:3/0:0), and LPC (18:1/0:0) were identified as representative metabolites that exhibited continuous upregulation or downregulation throughout the course of the disease (Figs. 5E–J). Collectively, these six metabolites showed pronounced and consistent change, indicating their potential as novel biomarkers for staging pulmonary inflammation. This was further supported by PCA, which revealed distinct clustering patterns corresponding to each disease progression stage based on these metabolites (Fig. S20). These findings validate the consistency between metabolic alterations and the RhB-PLL-based imaging stages.

### 3.6 FZJD exhibits significant efficacy in inhibiting the progression of pneumonia

**Fig. 6:**
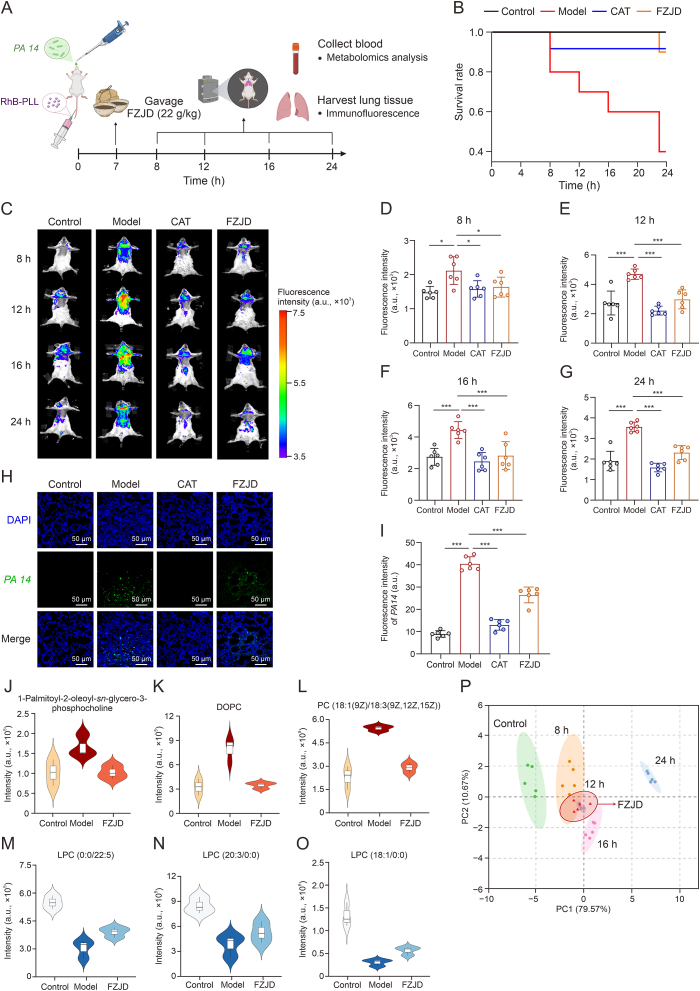
Evaluation of the therapeutic efficacy of Fuzheng Jiedu decoction (FZJD) in different stages of pneumonia based on rhodamine B-conjugated poly-L-lysine (RhB-PLL) nanoparticles (NPs) and metabolic biomarkers. (A) Schematic showing the experimental design of the animal study. (B) The survival curves of mice in the acute pulmonary inflammation (ALI) model in FZJD are presented. (C) *In vivo* fluorescence images of ALI model mice administered FZJD at each time point within 24 h are provided. (D–G) Histogram illustrating the relative fluorescence intensity (*n* = 6): 8 h (D), 12 h (E), 16 h (F), and 24 h (G). (H) Immunofluorescence images of *Pseudomonas aeruginosa* 14 (*PA 14*) strains distributed in the mouse lung. (I) Histogram depicts the relative fluorescence of *PA 14* strains (*n* = 6). (J–O) A violin plot of six representative differential metabolites in ALI model mice treated with FZJD is displayed (*n* = 3): 1-palmitoyl-2-oleoyl-*sn*-glycero-3-phosphocholine (J), 1,2-dioleoyl-*sn*-glycero-3-phosphocholine (DOPC) (K), phosphatidylcholine (PC) (18:1(9Z)/18:3(9Z,12Z,15Z)) (L), lysophosphatidylcholine (LPC) (0:0/22:5) (M), LPC (20:3/0:0) (N), and LPC (18:1/0:0) (O). (P) Principal component analysis (PCA) cluster analysis of six representative differential metabolites in each group is conducted. Data presented as mean ± standard deviation (SD). ^∗^*P* < 0.05 and ^∗∗∗^*P* < 0.001. CAT: i.p. ceftazidime 400 mg/kg and avibactam 100 mg/kg; DAPI: 4′,6-diamidino-2′-phenylindole.

Owing to the inherent complexity of TCM prescriptions, exploring the real-time effects of compatibility combinations on therapeutic outcomes *in vivo* remains a substantial challenge [33]. In this study, *in vivo* RhB-PLL imaging was employed to evaluate the therapeutic effects after intragastric administration of FZJD at 7 h post *PA 14* infection (Fig. 6A). Compared to the model group, which exhibited a 40% survival rate, both CAT and the FZJD treatment significantly increased survival rate to approximately 90% within 24 h (Fig. 6B). Both CAT and FZJD showed comparable efficacy in reducing pulmonary RhB-PLL fluorescence intensity from 8 to 24 h (Figs. 6C–G). Interestingly, immunohistochemical detection of *PA 14* strain in lung tissues showed that CAT markedly reduced bacterial burden. In contrast, only partial reduction was observed in the FZJD group, indicating that the alleviation of pneumonia by FZJD may not be solely bacteriostatic (Figs. 6H and I). Additionally, metabolomics analysis further confirmed that FZJD reversed the abnormal alterations in the six PC biomarkers (Figs. 6J–O). Subsequently, PCA based on these biomarkers was used to compare the FZJD and model groups across different stages. As depicted in Fig. 6P, FZJD treatment exhibited partial overlap with the 8-h model samples and complete overlap with the 12-h samples. These findings indicated that FZJD effectively alleviates pulmonary inflammation and stabilizes disease progression at the mild-to-moderate stages.

### 3.7 FZJD demonstrates compatibility in the treatment of the pneumonia process

**Fig. 7:**
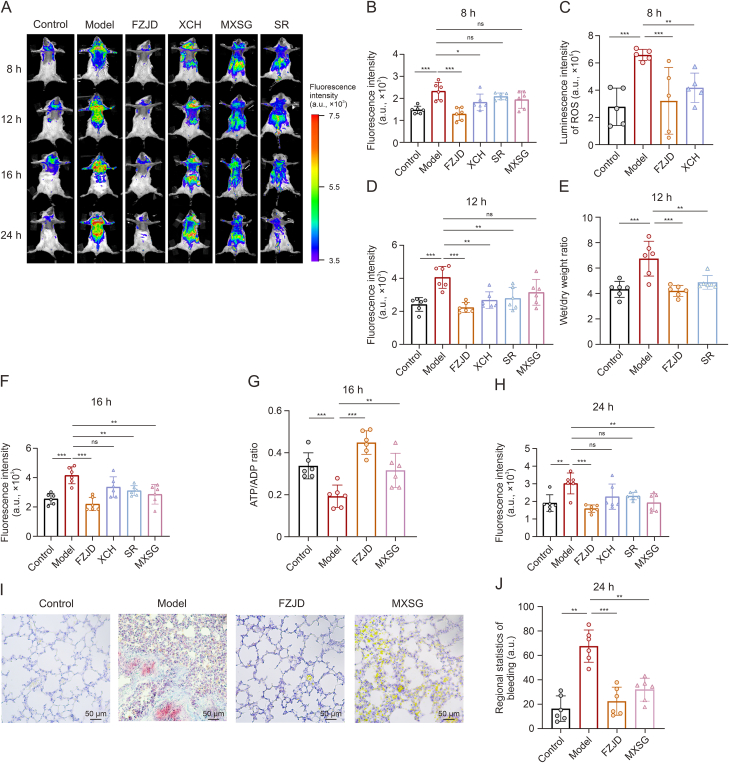
Different prescription combinations exhibit selective influences on the progression of pneumonia at various stages. (A) *In vivo* rhodamine B-conjugated poly-L-lysine (RhB-PLL) imaging of the administration of Fuzheng Jiedu decoction (FZJD) and its component formulas at four critical time points in pneumonia model mice (*n* = 6). (B) Statistics of RhB-PLL fluorescence intensity at 8 h. (C) Lung tissues reactive oxygen species (ROS)-responsive luminescence Cu-luminol (Cu-Lum)@nanoparticles (NPs) imaging analysis at 8 h. (D) Statistics of RhB-PLL fluorescence intensity at 12 h. (E) Wet/dry weight ratio of lung tissue at 12 h. (F) Statistics of RhB-PLL fluorescence intensity at 16 h. (G) Adenosine triphosphate/adenosine diphosphate (ATP/ADP) ratio detection in lung tissue at 16 h. (H) Statistics of RhB-PLL fluorescence intensity at 24 h. (I, J) The modified Masson's trichrome (MSB) staining (I) and corresponding bleeding pathological score analysis at 24 h (J). Data presented as mean ± standard deviation (SD). ^∗^*P* < 0.05, ^∗∗^*P* < 0.01, and ^∗∗∗^*P* < 0.001. ns: not significant. XCH: Xiaochaihu decoction; MXSG: Maxingshigan decoction; SR: Sanren decoction.

Due to FZJD being formed through the integration of three classical formulas, XCH, SR, and MXSG, the roles of these simplified prescriptions were investigated using RhB-PLL NPs to examine their characteristics in real-time at various stages *in vivo*. As shown in Fig. 7A, compared with the model group, FZJD demonstrated significant effects throughout the entire disease course. However, the functional characteristics differed among the various formulations simplified from the FZJD. The impact of XCH was observed from 8 to 12 h, suggesting its efficacy during the mild to moderate stage of pulmonary inflammation. However, its efficacy in halting disease progression was limited at later stages (Fig. 7B). The anti-inflammatory effect of XCH was supported by its ability of inhibition of oxidative stress, as evidenced by ROS-responsive luminescence detected with Cu-Lum@NPs probes (Fig. 7 C). Different from XCH, SR treatment showed significantly reduced RhB-PLL fluorescence especially at 12 h, accompanied by a marked reduction in pulmonary edema (Figs. 7D and E). These findings suggest that SR may prevent the progression of edema in the moderate to severe stages by stabilizing the structure and decreasing the permeability of epithelial or endothelial cells.

From 16 to 24 h, a relatively smaller change in fluorescence was observed in the MXSG group (Fig. 7F). Both MXSG and FZJD significantly increased the ATP/ADP ratios in lung tissue, suggesting a protective effect on mitochondrial function during the severe and critical stages of pneumonia (Figs. 7G and H). Furthermore, MSB staining indicated that MXSG mitigated microvascular damage caused by capillary hemorrhage in the later stages (Figs. 7I and J). In summary, RhB-PLL spatiotemporal imaging clearly visualized and characterized the stage-specific therapeutic effects of FZJD and its simplified prescriptions.

### 3.8 The safety evaluation of RhB-PLL NPs

To exclude potential NP-related effects on living organisms, a comprehensive series of safety assessment experiments were conducted. Following a single intravenous administration of RhB-PLL at a dose of 20 mg/kg in mice, no significant changes in body weight were observed during the 14-day observation period (Fig. S21A). In addition, no notable alterations were detected in the organ indices of major organs (Fig. S21B). Liver-related biomarkers, including aspartate aminotransferase (AST), alanine aminotransferase (ALT), total protein (TP), and ALB, remained within normal reference ranges (Fig. S21C). Similarly, kidney function indicators, such as urea and creatinine (Cre), remained within normal limits, with no abnormalities detected (Fig. S21D). Histological examination of major organs by H&E staining revealed no abnormalities (Fig. S21E). Collectively, these findings indicated that RhB-PLL exhibits relatively good biocompatibility and safety, making it suitable for evaluating the progression of pulmonary inflammation *in vivo*.

## 4 Discussion

Traditional pneumonia staging methods include clinical presentation-based staging, which categorizes pneumonia severity by symptoms and signs but is somewhat subjective. Microbiological testing-based staging identifies pathogens through sputum tests to guide treatment but has limitations when used as the sole staging method [34]. Blood test-based staging measures indicators such as white blood cell counts and procalcitonin to evaluate inflammation and damage but lacks specificity due to multiple influencing factors [35]. Chest imaging-based staging assesses the extent and severity of inflammation via X-rays or computed tomography (CT) scans [36]. In this study, noticeable liver fluorescence was observed, which was primarily attributed to Kupffer cells that efficiently engulf particles ranging from 10 to 200 nm [37]. The significantly enhanced fluorescence in the injured lung suggested that, beyond passive hepatic uptake, RhB-PLL demonstrated a preferential accumulation in inflamed pulmonary tissues due to increased vascular permeability and electrostatic attraction-driven mitochondrial targeting.

Precision medicine aims to match therapies to specific pathological progressions, thereby maximizing patient benefit. Stage-based treatment is essential for optimizing the management of pneumonia. For instance, official clinical practice guidelines have been issued for the diagnosis and treatment of adults with community-acquired pneumonia (CAP), hospital-acquired pneumonia, and ventilator-associated pneumonia (VAP) [38]. In early-stage CAP, β-lactam monotherapy (e.g., amoxicillin) achieves similar 30-day mortality rates compared to broad-spectrum regimens while reducing the risk of antibiotic resistance. In contrast, late-stage VAP caused by multidrug-resistant pathogens requires targeted combinations such as ceftazidime-avibactam, which demonstrates superior efficacy against extended-spectrum beta-lactamase (ESBL)-producing *Enterobacteriaceae* compared to carbapenems [39]. This highlights the critical importance of accurately assessing disease progression. Nonetheless, the lack of adequate characterization methods impedes the comprehension of pharmacological effects of TCM and the implementation of precise diagnosis and treatment strategies. Clinically, pneumonia is typically staged using score systems such as the pneumonia severity index (PSI) and confusion, urea, respiratory rate, blood pressure, and age ≥ 65 years (CURB-65). While PSI offers comprehensive risk stratification, it is time-consuming and less suited for real-time assessment [40]. CURB-65 is simpler but still relies on clinical and laboratory parameters, limiting its applicability in animal models. In this study, we developed RhB-PLL NPs that enable real-time, non-invasive visualization of pneumonia progression by sensing vascular permeability and mitochondrial dysfunction. Based on fluorescence dynamics, pneumonia was classified into four stages: mild, moderate, severe, and critical, and validated by histological and biochemical markers. Compared to clinical scoring systems, the RhB-PLL-based strategy provides a feasible and mechanistically relevant approach for pneumonia staging in preclinical models.

TCM has demonstrated promising efficacy in the treatment of pneumonia. Unlike antibiotic therapy, which primarily alleviates pulmonary inflammation and improves survival rates primarily by reducing bacterial load through potent antibacterial effects, TCM exerts multidimensional immune regulation and tissue protection through synergistic mechanisms [41]. Here, RhB-PLL *in vivo* imaging provided a visual analytical approach for the multicomponent drug system derived from FZJD, elucidating the pharmacological roles of its simplified prescriptions at different disease stages. During the mild to moderate stages, PAMPs activate Toll-like receptor 4 (TLR-4). This activation subsequently upregulates the expression of vascular endothelial selectin (E-selectin), leukocyte selectin (L-selectin), vascular cell adhesion molecule-1 (VCAM-1), and intercellular adhesion molecule-1 (ICAM-1). As a result, this signaling cascade enhances leukocyte adhesion, facilitates inflammatory cell infiltration, and stimulates the innate immune response. At this stage, XCH effectively suppresses inflammatory cell infiltration and alleviates oxidative stress. In the severe stage, SRC proto-oncogene (SRC) kinase phosphorylates caveolin-1 and VE-cadherin in vascular endothelial cell microdomains, resulting in plasma ALB exudation, edema, and respiratory issues [42]. SR decoction significantly improved the dry-to-wet weight ratio and reduced edema at both the moderate and severe stages. In the critical stage, except for NETs mediating platelet aggregation, activated SRC leads to vascular endothelial cell turnover, basement membrane exposure, and microvascular complications such as hemorrhage and thrombosis, which may progress to disseminated intravascular coagulation (DIC) [43]. MXSG effectively mitigated vascular leakage and platelet thrombosis formation, and significantly enhanced mitochondrial function. As anticipated, the combination formula FZJD exhibits remarkable efficacy in alleviating pneumonia progression across all stages. Rather than solely targeting pathogen elimination, it modulates host immune responses and restores physiological equilibrium, thereby promoting comprehensive recovery.

The identification of reliable biomarkers is crucial for the diagnosis, prognosis, and treatment of pulmonary inflammation. Recently, gene-expression-based diagnostic approaches have shown promise for pneumonia detection [44]. For example, RNA sequencing of blood samples from pediatric pneumonia patients identified a five-transcript signature (genes *FAM20A*, *BAG3*, *TDRD9*, *MXRA7*, and *KLF14*) that effectively differentiates bacterial from viral pneumonia [45]. Bioinformatics analyses have also identified key differentially expressed genes, such as *CCL4*, *TIMP1*, *ICAM1*, *PLAUR*, and *CTSB*, as potential biomarkers for pneumonia [46]. Metabolomics analysis represents a robust approach for evaluating therapeutic efficacy and prognosis after drug intervention [47]. Notably, syndrome-based metabolomics is an essential tool for assessing TCM efficacy [48]. By analyzing the dynamic fluorescence trends of RhB-PLL NPs, disease progression was systematically classified into four stages: mild, moderate, severe, and critical. Based on the screening of characteristic metabolic markers for each stage, six potential biomarkers were identified as candidate indicators for pneumonia: three PCs and three LPCs, which exhibit stage-specific variations in PC metabolism. Monitoring these biomarkers across treatment groups further demonstrated that FZJD treatment significantly halted the pneumonia progression at the mild to moderate stages. Metabolomics cluster analysis based on these biomarkers not only enhances the diagnostic accuracy of RhB-PLL imaging, but also shows potential for differentiating infections stages, guiding personalized treatments, and monitoring treatment efficacy.

## 5 Conclusion

The heterogeneity in lesion progression during infection underscores the urgent need for advanced methodologies for precise *in situ* observation of biological processes and host responses. This study demonstrated that multimodal RhB-PLL NPs enable real-time visualization of pneumonia progression, thereby enhancing our understanding of disease staging and the efficacy of intervention in multicomponent drug systems. Additionally, the findings confirmed the efficacy of stage-based interventions in pneumonia treatment and highlighted the advantages of formulation compatibility. This result provide robust scientific evidence for the sequential and synergistic application of TCM and facilitate more precise administration in pneumonia treatment.

## CRediT authorship contribution statement

**Man Zhang:** Writing – review & editing, Writing – original draft, Visualization, Validation, Data curation. **Shanshan Zhai:** Writing – original draft, Methodology, Investigation, Formal analysis. **He Gao:** Methodology, Investigation, Formal analysis. **Tong Sun:** Methodology, Investigation, Formal analysis. **Kaixin Liu:** Methodology, Investigation. **Wenshuang Wang:** Validation, Resources, Methodology. **Yuanyuan Hou:** Writing – review & editing, Resources, Funding acquisition, Data curation, Conceptualization. **Gang Bai:** Supervision, Resources, Project administration, Conceptualization.

## Declaration of competing interest

The authors declare that they have no known competing financial interests or personal relationships that could have appeared to influence the work reported in this paper.

## References

[1] Quinton L.J., Walkey A.J., Mizgerd J.P. (2018). Integrative physiology of pneumonia. Physiol. Rev..

[2] Qian C., Zhu W., Wang J. (2024). Cyclic-di-GMP induces inflammation and acute lung injury through direct binding to MD2. Clin. Transl. Med..

[3] Nathan C. (2002). Points of control in inflammation. Nature.

[4] Nathan C. (2006). Neutrophils and immunity: challenges and opportunities. Nat. Rev. Immunol..

[5] Stark K., Massberg S. (2021). Interplay between inflammation and thrombosis in cardiovascular pathology. Nat. Rev. Cardiol..

[6] Baedorf-Kassis E., Murn M., Dzierba A.L. (2024). Respiratory drive heterogeneity associated with systemic inflammation and vascular permeability in acute respiratory distress syndrome. Crit. Care Lond. Engl..

[7] Ren H., He Y., Liang J. (2019). Role of liposome size, surface charge, and PEGylation on rheumatoid arthritis targeting therapy. ACS Appl. Mater. Interfaces.

[8] Janiak F.K., Bartel P., Bale M.R. (2022). Non-telecentric two-photon microscopy for 3D random access mesoscale imaging. Nat. Commun..

[9] Sun Z., Tong G., Liu Y. (2022). Dual function of a *in vivo* albumin-labeling tracer for assessment of blood perfusion and vascular permeability in peripheral arterial disease by PET. Front. Cardiovasc. Med..

[10] Mankoff D.A., Pantel A. (2020). PET molecular imaging as a tool for precision oncology. Radiology.

[11] Schuster D.P., Stark T., Stephenson J. (2002). Detecting lung injury in patients with pulmonary edema. Intensive Care Med..

[12] Ogasawara N., Suga K., Karino Y. (2002). Perfusion characteristics of radiation-injured lung on Gd-DTPA-enhanced dynamic magnetic resonance imaging, Investig. Radiol..

[13] Dafni H., Cohen B., Ziv K. (2005). The role of heparanase in lymph node metastatic dissemination: dynamic contrast-enhanced MRI of Eb lymphoma in mice. Neoplasia.

[14] Short K.R., Kroeze E.J.B.V., Fouchier R.A.M. (2014). Pathogenesis of influenza-induced acute respiratory distress syndrome. Lancet Infect. Dis..

[15] Nguyen D.D., Luo L.J., Yang C.J. (2023). Highly retina-permeating and long-acting resveratrol/metformin nanotherapeutics for enhanced treatment of macular degeneration. ACS Nano.

[16] Gao H., Sun T., Wang W. (2024). Self-illuminating copper-luminol coordination polymers for bioluminescence imaging of oxidative damage. Anal. Chem..

[17] Yang C.J., Nguyen D.D., Lai J.Y. (2023). Poly(l-histidine)-mediated on-demand therapeutic delivery of roughened ceria nanocages for treatment of chemical eye injury. Adv. Sci..

[18] Wang S., Wang R., Chen J. (2023). Controlled extracellular vesicles release from aminoguanidine nanoparticle-loaded polylysine hydrogel for synergistic treatment of spinal cord injury. J. Control. Release.

[19] Wei J., Guo X., Wang Y. (2024). Realizing real-time optical molecular imaging in peripheral nerve tissue via rhodamine B. Front. Med..

[20] Ma S., Cong Z., Wei J. (2022). Pulmonary delivery of size-transformable nanoparticles improves tumor accumulation and penetration for chemo-sonodynamic combination therapy. J. Control. Release.

[21] Rejman J., Oberle V., Zuhorn I.S. (2004). Size-dependent internalization of particles via the pathways of clathrin- and caveolae-mediated endocytosis. Biochem. J..

[22] Dorn G.W., Kitsis R.N. (2015). The mitochondrial dynamism-mitophagy-cell death interactome: multiple roles performed by members of a mitochondrial molecular ensemble. Circ. Res..

[23] Zielonka J., Joseph J., Sikora A. (2017). Mitochondria-targeted triphenylphosphonium-based compounds: syntheses, mechanisms of action, and therapeutic and diagnostic applications. Chem. Rev..

[24] Nava Lauson C.B., Tiberti S., Corsetto P.A. (2023). Linoleic acid potentiates CD8^+^ T cell metabolic fitness and antitumor immunity. Cell Metab..

[25] Agudelo C.W., Samaha G., Garcia-Arcos I. (2020). Alveolar lipids in pulmonary disease. A review. Lipids. Heal. Dis..

[26] Zong Y., Li H., Liao P. (2024). Mitochondrial dysfunction: mechanisms and advances in therapy. Signal Transduct. Target. Ther..

[27] Biondi B., Palmieri E.A., Lombardi G. (2002). Effects of subclinical thyroid dysfunction on the heart. Ann. Intern. Med..

[28] Yao M., Ma J., Wu D. (2023). Neutrophil extracellular traps mediate deep vein thrombosis: from mechanism to therapy. Front. Immunol..

[29] Spadaro S., Park M., Turrini C. (2019). Biomarkers for acute respiratory distress syndrome and prospects for personalised medicine. J. Inflamm. Lond. Engl..

[30] Cañadas O., Olmeda B., Alonso A. (2020). Lipid-protein and protein-protein interactions in the pulmonary surfactant system and their role in lung homeostasis. Int. J. Mol. Sci..

[31] Batsika C.S., Gerogiannopoulou A.D., Mantzourani C. (2021). The design and discovery of phospholipase A_2_ inhibitors for the treatment of inflammatory diseases. Expert Opin. Drug Discov..

[32] Law S.H., Chan M., Marathe G.K. (2019). An updated review of lysophosphatidylcholine metabolism in human diseases. Int. J. Mol. Sci..

[33] Guo X., An S., Bao F. (2023). Challenges and perspectives in target identification and mechanism illustration for Chinese medicine, Chin. J. Integr. Med..

[34] Liapikou A., Cillóniz C., Torres A. (2019). Emerging strategies for the noninvasive diagnosis of nosocomial pneumonia. Expert Rev. Anti Infect. Ther..

[35] Franquet T. (2001). Imaging of pneumonia: trends and algorithms. Eur. Respir. J..

[36] Slika B., Dornaika F., Merdji H. (2024). Lung pneumonia severity scoring in chest X-ray images using transformers. Med. Biol. Eng. Comput..

[37] Ngo W., Ahmed S., Blackadar C. (2022). Why nanoparticles prefer liver macrophage cell uptake *in vivo*. Adv. Drug Deliv. Rev..

[38] Metlay J.P., Waterer G.W., Long A.C. (2019). Diagnosis and treatment of adults with community-acquired pneumonia. An official clinical practice guideline of the American thoracic society and infectious diseases society of America. Am. J. Respir. Crit. Care Med..

[39] Magill S.S., O'Leary E., Janelle S.J. (2018). Changes in prevalence of health care-associated infections in U.S. hospitals, N Engl J. Med.

[40] Fine M.J., Auble T.E., Yealy D.M. (1997). A prediction rule to identify low-risk patients with community-acquired pneumonia. N Engl J. Med.

[41] Zou Q., Chen Y., Qin H. (2023). The role and mechanism of TCM in the prevention and treatment of infectious diseases. Front. Microbiol..

[42] Sartori C., Matthay M.A. (2002). Alveolar epithelial fluid transport in acute lung injury: new insights. Eur. Respir. J..

[43] Gaertner F., Massberg S. (2019). Patrolling the vascular borders: platelets in immunity to infection and cancer. Nat. Rev. Immunol..

[44] Xie J., Li Y., Wang M. (2022). Diagnostic and prognostic value of dysregulated miR-10a-3p in patients with severe pneumonia. J. Inflamm. Res..

[45] Viz-Lasheras S., Gómez-Carballa A., Pardo-Seco J. (2025). A 5-transcript signature for discriminating viral and bacterial etiology in pediatric pneumonia. iScience.

[46] Vastrad B., Vastrad C., A T. (2020). Identification of potential mRNA panels for severe acute respiratory syndrome coronavirus 2 (COVID-19) diagnosis and treatment using microarray dataset and bioinformatics methods. 3 Biotech.

[47] Zhou Y., Wei Y., Wang J. (2025). Efficacy and metabolomic analysis of the pneumonia compound formulation against community-acquired pneumonia: an observational controlled before-after clinical trial. BMC Infect. Dis..

[48] Wang M., Yin F., Kong L. (2024). Chinmedomics: a potent tool for the evaluation of traditional Chinese medicine efficacy and identification of its active components. Chin. Med..

