# Supplementary material for: Classification of pulmonary inflammation stages and assessment of multicomponent drug intervention based on spatiotemporal imaging variations utilizing RhB-conjugated poly-L-lysine nanoparticles
Source: J Pharm Anal. 2026 Feb 9;16(7):101582. doi: 10.1016/j.jpha.2026.101582 (PMC13382254; doi:10.1016/j.jpha.2026.101582)
Supplement: Multimedia component 1 [file mmc1.docx]

**Supplementary Data**

**Classification of pulmonary inflammation stages and assessment of multicomponent drug intervention** **based on** **spatiotemporal imaging variations utilizing RhB-conjugated poly-L-lysine nanoparticles**

Man Zhang**^1^**, Shanshan Zhai**^1^**, He Gao, Tong Sun, Kaixin Liu, Wenshuang Wang, Yuanyuan Hou^**^, Gang Bai^*^

*State Key Laboratory of Medicinal Chemical Biology, College of Pharmacy and Tianjin Key Laboratory of Molecular Drug Research, Nankai University, Tianjin, 300353, China*

* Corresponding author.

Gang Bai

State Key Laboratory of Medicinal Chemical Biology, College of Pharmacy and Tianjin Key Laboratory of Molecular Drug Research, Nankai University, Tianjin, 300353, China

**Corresponding author.

*E-mail address*: [houyy@nankai.edu.cn](mailto:houyy@nankai.edu.cn) (Y. Hou), [gangbai@nankai.edu.cn](mailto:gangbai@nankai.edu.cn) (G. Bai)

^1^Both authors contributed equally to this work.

**1. The decoction process of traditional Chinese medicine decoctions and HPLC identification.**

1. **Purchase of medicinal materials and reference materials:**

Ephedrine hydrochloride (171241-201508) was sourced from National Institutes for Food and Drug Control (Shanghai, China; purity: HPLC ≥ 98%). Lobetyolin (CFS202402) were purchased from Chemfaces Biotechnology Co., Ltd. (Wuhan, China; purity: HPLC ≥ 98%). Liquiritigenin (PS1073-0020) were purchased from Chengdu Push Biotechnology Co., Ltd. (Chengdu, China; purity: NC ≥ 98%). Saikosaponin A (B20146), amygdalin (S30650) and polydatin (S31397) were purchased from Shanghai Yuanye Biotechnology Co., Ltd. (Shanghai, China; purity: HPLC ≥ 98%). Coixol (ST80220120), apigenin (ST00410120) and wogonin (ST01710120) were purchased from Shanghai Standard Technology Co., Ltd (Shanghai, China; purity: HPLC ≥ 98%). Herbs in Fuzheng Jiedu decoction (FZJD), Xiaochaihu decoction (XCH), Sanren decoction (SR) and Maxingshigan decoction (MXSG) were purchased from Beijing Tong Ren Tang Co., Ltd (Beijing, China).

**2) HPLC Testing Conditions:**

Chromatographic instruments: LC-20AT high performance liquid chromatograph, SPD-20A detector, Shimadzu Corporation, Japan.

Chromatographic conditions: Cosmosil HPLC®C18 chromatographic column (250 mm×4.6 mm, 5 μm); Mobile phase - Pure water solution (A) - acetonitrile (B) Gradient elution: 0-6 min, 5% - 10%B; 6 to 60 minutes, 10% to 25%B; 60-65 minutes, 25% - 30%B; 65-70 minutes, 30% - 50%B; 70-80 minutes, 50% - 90%B; 80-100 minutes, 90% - 100%B; The temperature is 33℃. The injection volume is 10 μL; The volumetric flow rate is 1.0 mL/min and the detection wavelength is 210 nm.

The preparation of reference substance solutions: Take ephedrine hydrochloride reference substance, amygdalin reference substance, codonopsis pilosula reference substance, polygonum cuspidatum reference substance, coix seed reference substance, glycyrrhizin reference substance, bupleuronin A reference substance, apigenin reference substance and wogonin reference substance. Take an appropriate amount, accurately weigh it, and add methanol to prepare mixed reference substance solutions with mass fractions of 50 μg/mL respectively.

**3) Preparation of the test solution:**

Take 20 g of Codonopsis pilosula, 15 g of Bupleurum chinense, 15 g of Scutellaria baicalensis, 9g of honey-fried Ephedra, 9g of stir-fried bitter almonds, 30 g of raw gypsum, 15 g of Polygonum cuspidatum, 20 g of Coix seed, 10 g of cardamom seed, 10 g of Pinellia ternata, and 6 g of honey-fried licorice. Boil them twice. For the first time, add 10 times the amount of water and boil for 1 hour. Then filter. For the second time, add 10 times the amount of water and boil for 1 hour. Filter, combine the filtrates, and reduce the pressure to concentrate the filtrates into a paste. Take 0.5g of the concentrated compound paste, add 50 mL of 75% methanol, ultrasonically treat for 30 minutes, centrifuge at 12,000 rpm for 10 minutes, and take the supernatant to prepare the compound sample of the Fuzheng Jiedu Formula. The processing of other samples of Xiaochaihu Decoction, Sanren Decoction and Maxing Shigan Decoction is the same as above.


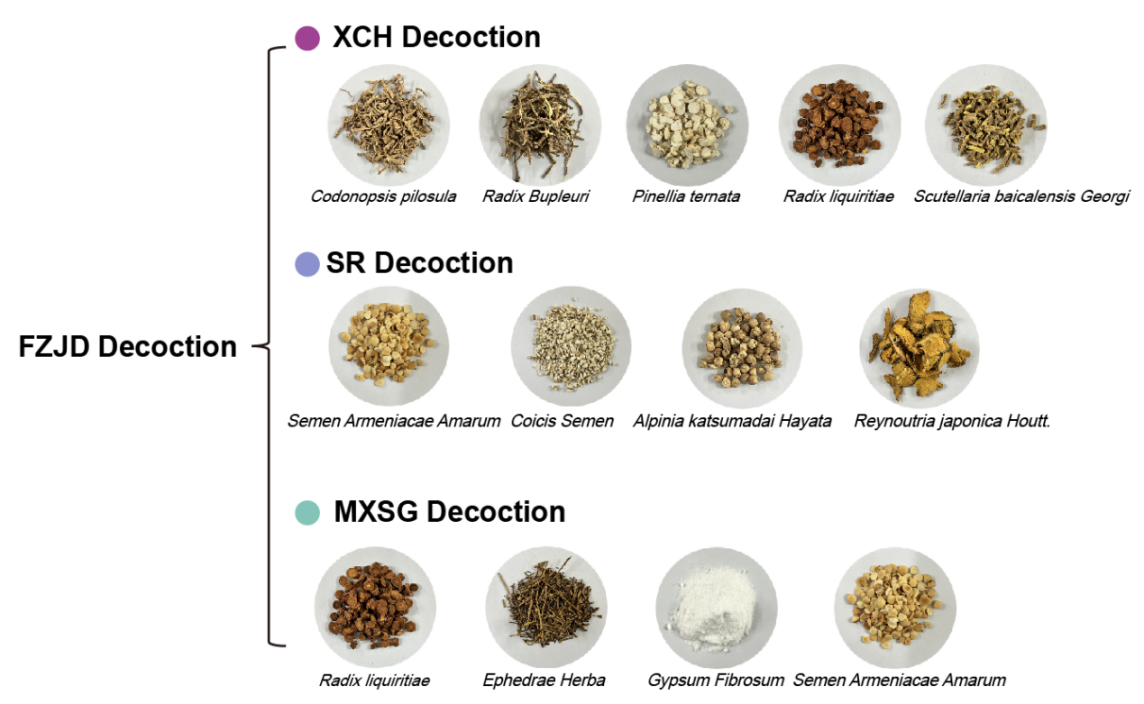


**Fig. S1.** Schematic diagram of the composition of Fuzheng Jiedu decoction (FZJD), Xiaochaihu decoction (XCH), Sanren decoction (SR), and Maxingshigan decoction (MXSG).

**
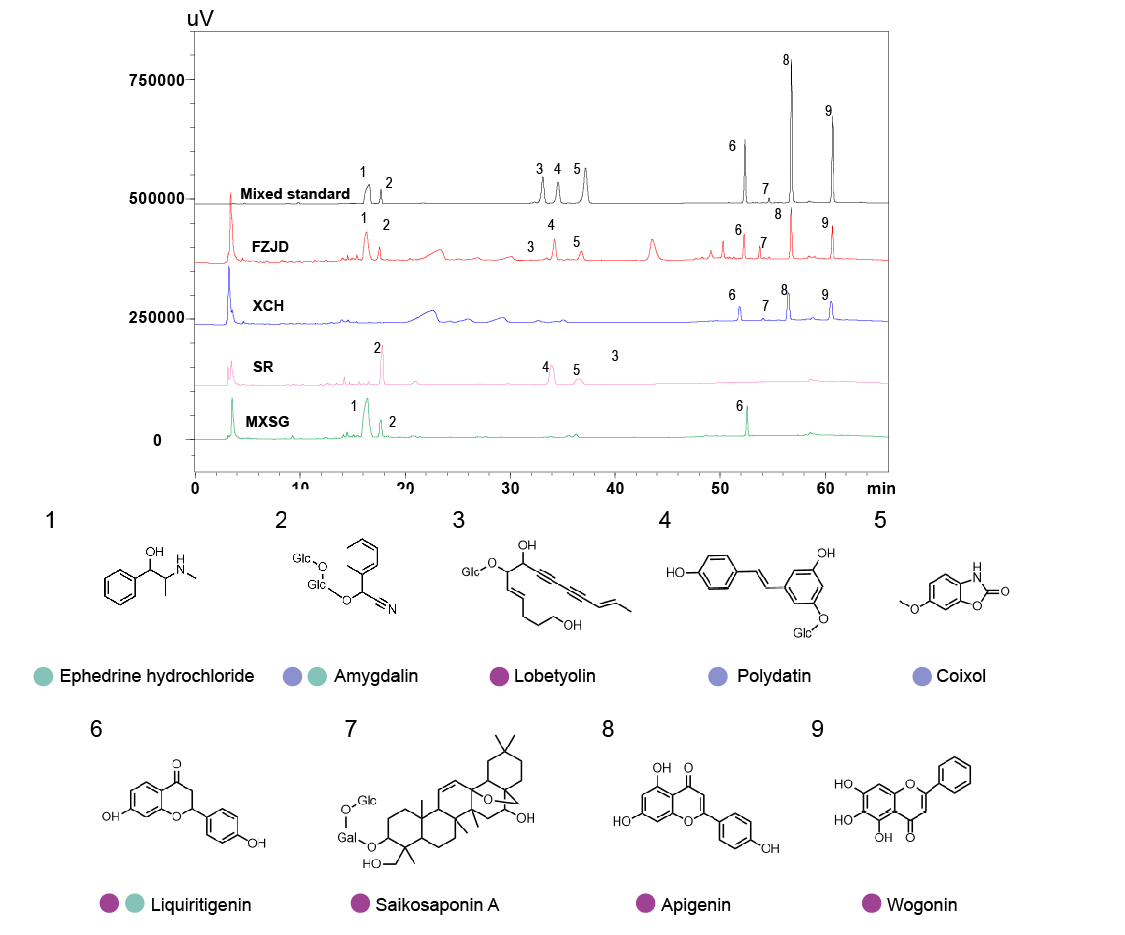
**

**Fig. S2.** High Performance Liquid Chromatography (HPLC) identification of representative components of Fuzheng Jiedu decoction (FZJD), Xiaochaihu decoction (XCH), Sanren decoction (SR), and Maxingshigan decoction (MXSG).


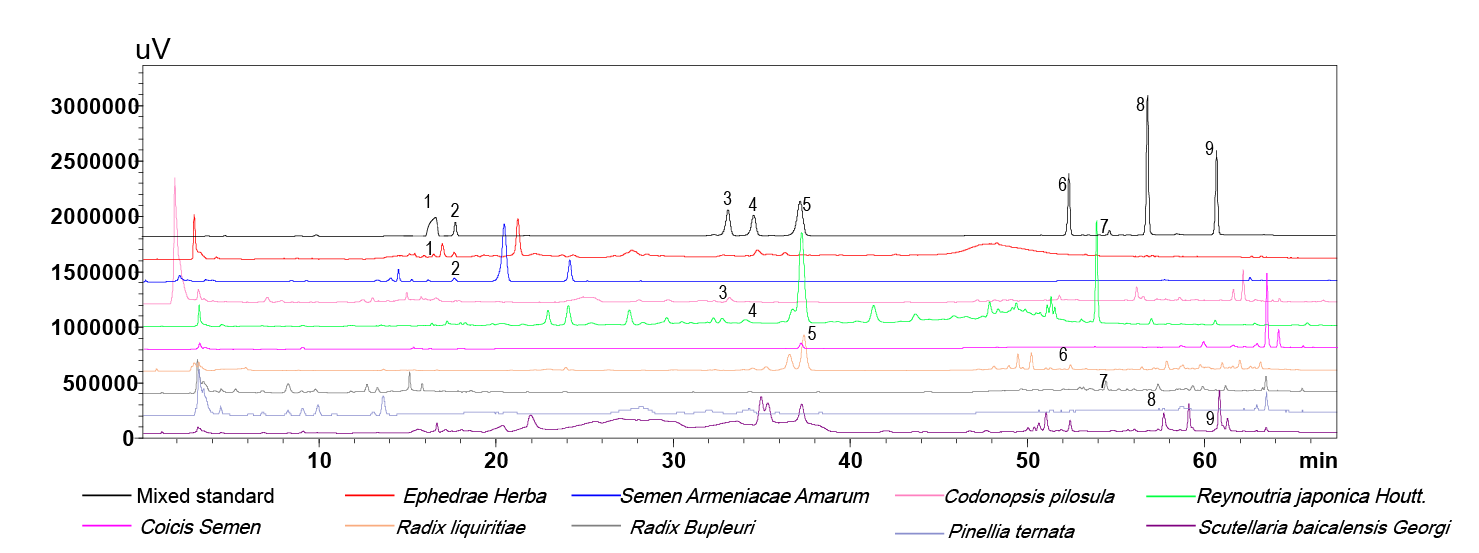


**Fig. S3.** High Performance Liquid Chromatography (HPLC) identification of representative components of each medicinal material in Fuzheng Jiedu (FZJD) Decoction


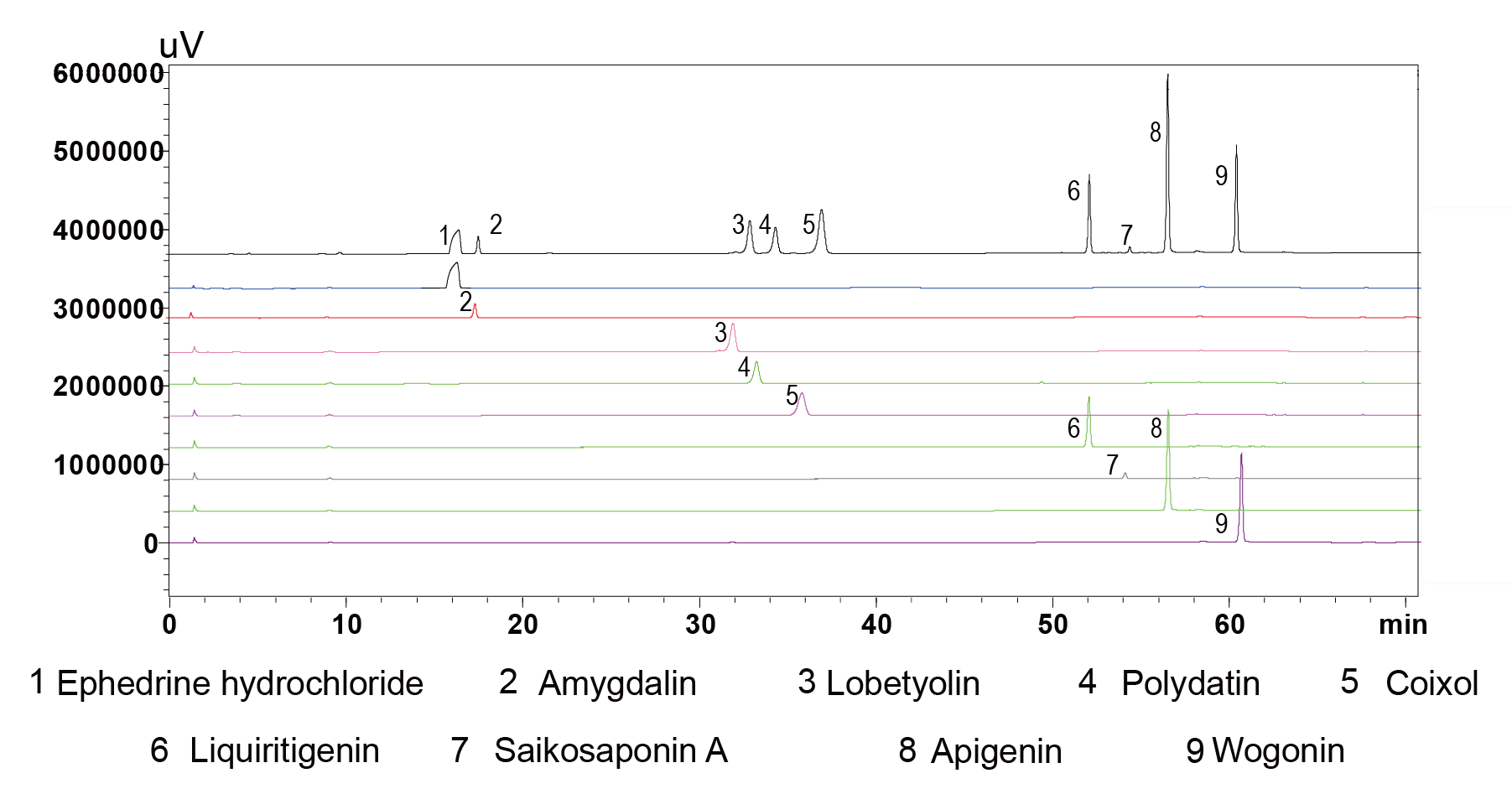


**Fig. S4.** Mixed reference substances and individual reference substances High Performance Liquid Chromatography (HPLC)

**2. Structural identification of Rhodamine B-conjugated poly-L-lysine (RhB-PLL).**

The two products in the synthetic route (Figure 1A) were detected by NMR, HMRS and LC-MS.

The test results of the one-step product NHS-RhB are as follows: ^1^H NMR (400 MHz, CDCl3) δ 8.41 (d, J = 7.9 Hz, 1H), 7.96 (t, J = 7.7 Hz, 1H), 7.82 (t, J = 7.7 Hz, 1H), 7.45 (d, J = 7.6 Hz, 1H), 7.07 (d, J = 9.3 Hz, 2H), 6.84 (d, J = 10.7 Hz, 4H), 3.62 (d, J = 7.3 Hz, 8H), 2.65 (s, 4H), 1.31 (t, J = 7.0 Hz, 12H). ^13^C NMR (101 MHz, CDCl3) δ 12.74, 25.60, 46.16, 46.30, 76.84, 77.16, 77.36, 77.48, 96.68, 113.48, 114.52, 125.43,130.83, 131.05, 131.13, 131.89, 135.01, 155.73, 157.89, 168.94, 172.88, 172.91.


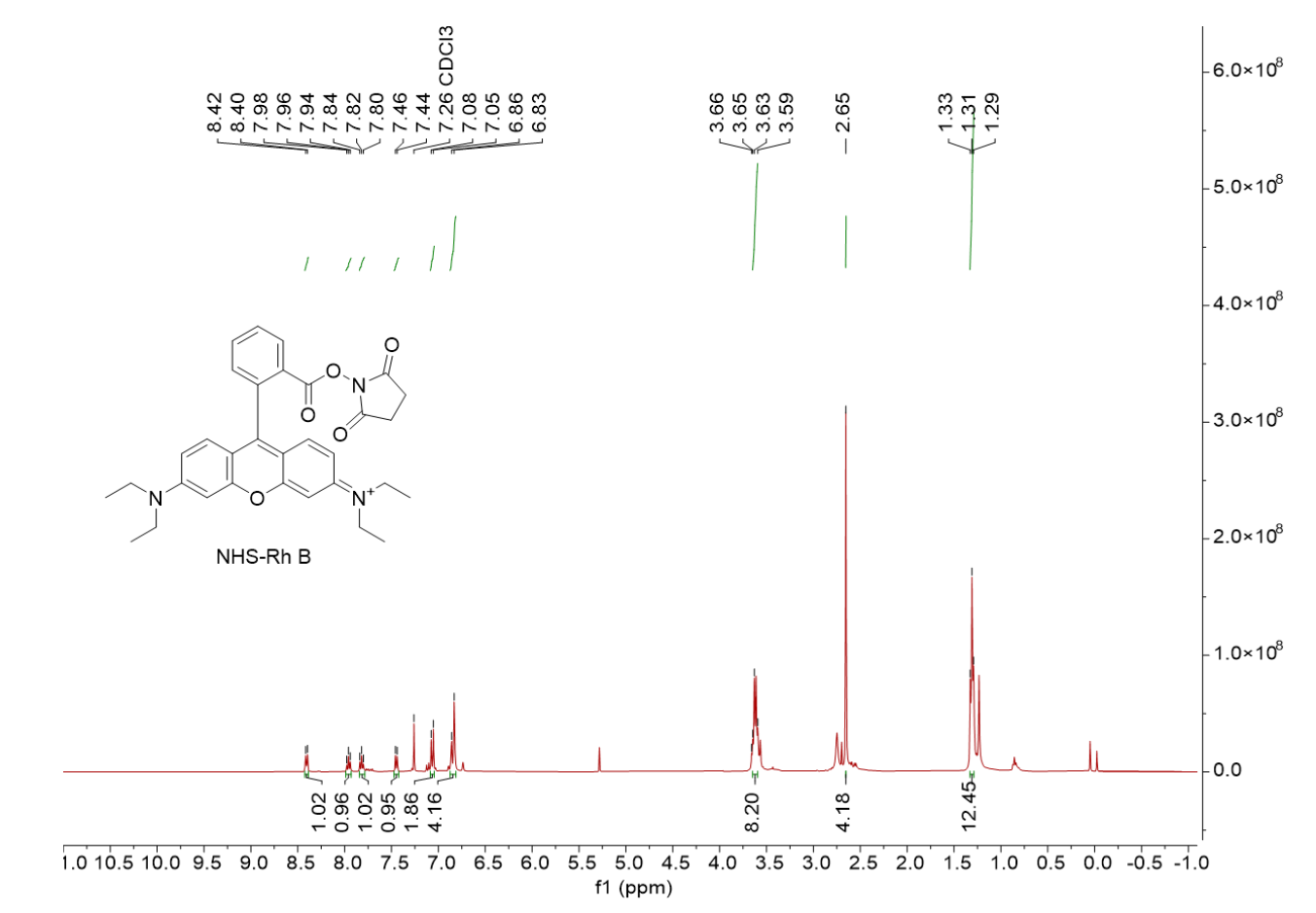


**Fig. S5.** ^1^H Nuclear Magnetic Resonance (NMR) of NHS Rhodamine B


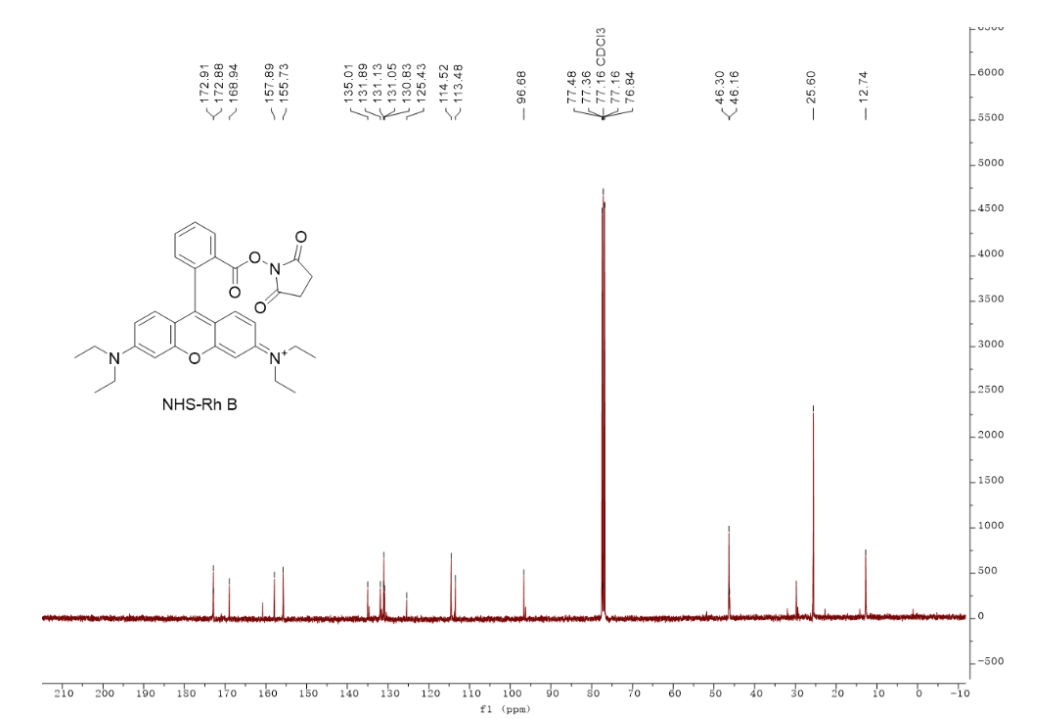


**Fig. S6.** ^13^C Nuclear Magnetic Resonance (NMR) of NHS Rhodamine B


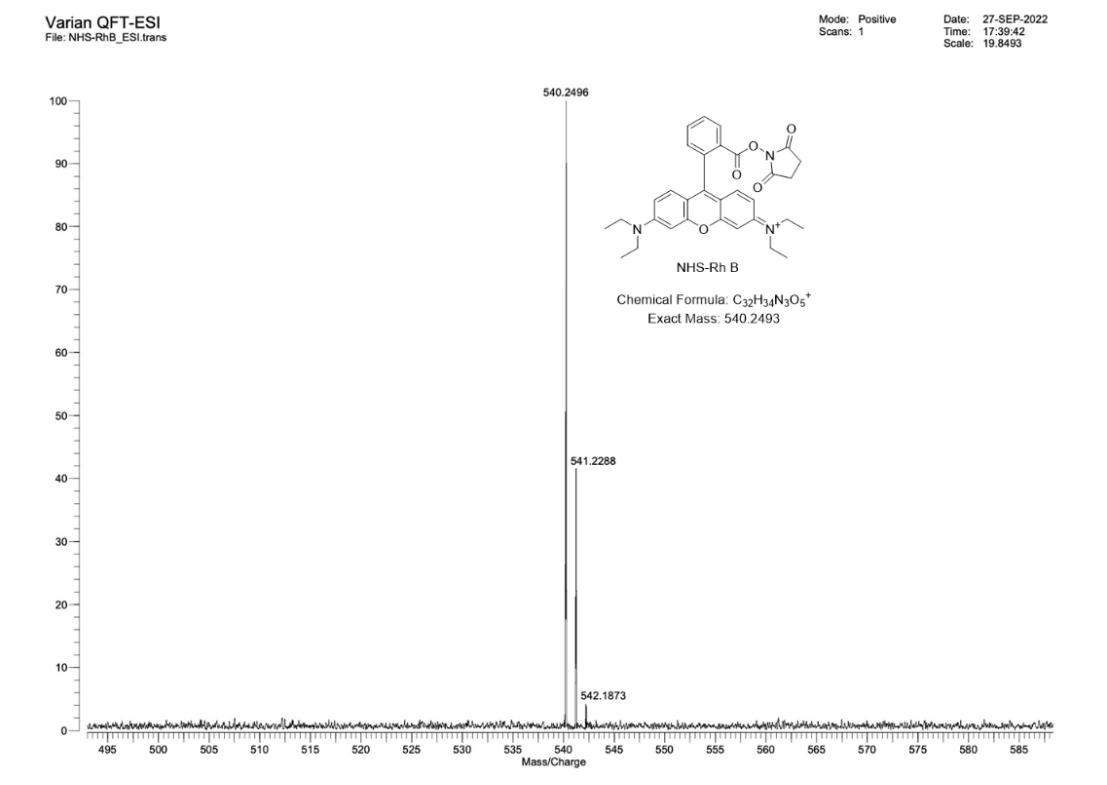


**Fig. S7.** High-Resolution Mass Spectrometry (HRMS) of NHS Rhodamine B

The results of the hydrogen spectrum of the two-step product show that between the chemical shift 1.0-2.0, it is mainly the characteristic peak of -H of the -CH2 group in the polylysine structure, suggesting the chain structure of polylysine. The chemical shift between 6.5 and 8.5 is mainly the -H on the RhB benzene ring group, and the single peak near the chemical shift 1.0. Comprehensive detection indicates that the grafting of RhB-PLL was successful.


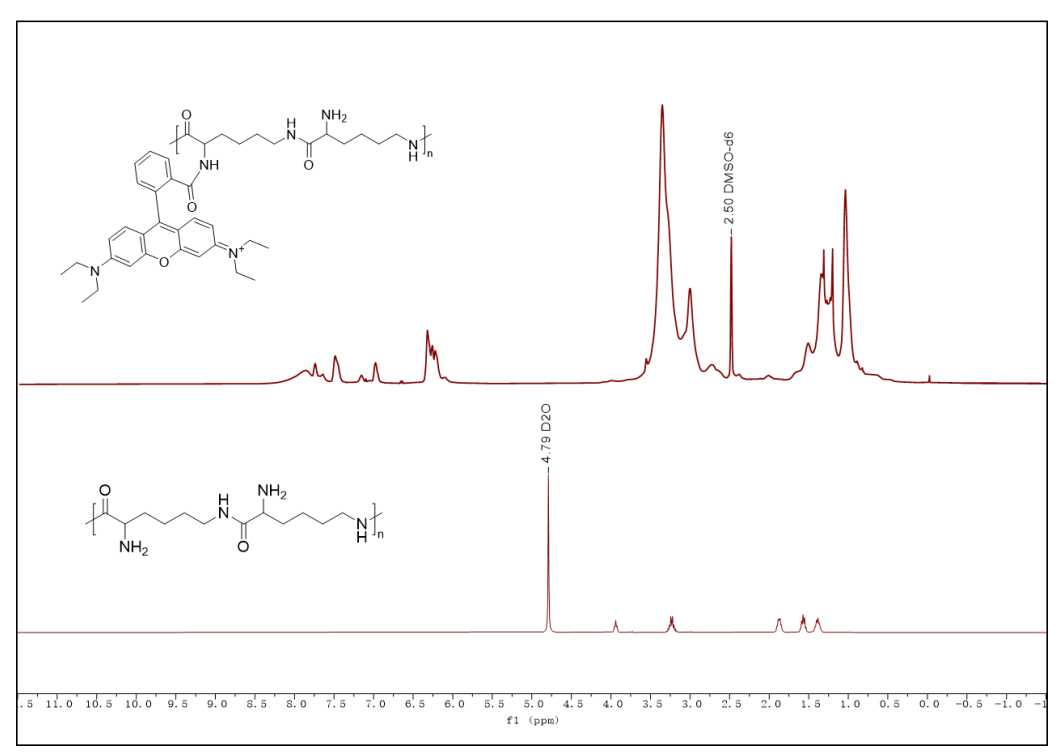


**Fig. S8.** ^1^H Nuclear Magnetic Resonance (NMR)of Rhodamine B-conjugated poly-L-lysine (RhB-PLL) and polylysine


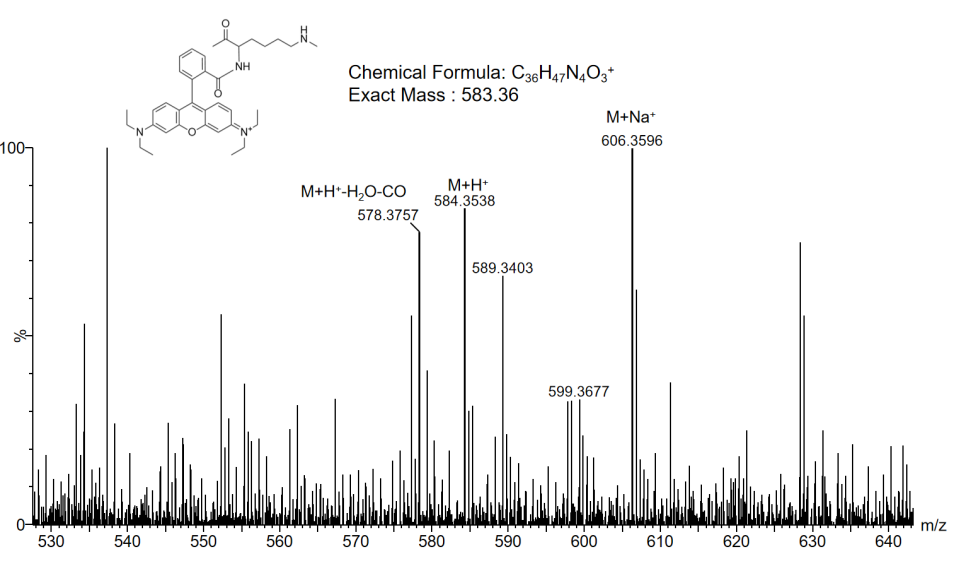


**Fig. S9.** Liquid Chromatography-Mass Spectrometry (LC-MS) of Rhodamine B-conjugated poly-L-lysine (RhB-PLL)

**3. Calculation of the number of RhB grafts in RhB-PLL**

Self-assembly of nanoparticles was conducted using an equivalent ratio of PLL:NHS-RhB = 1:5. Subsequently, the reaction solution was transferred to a dialysis bag (molecular weight cutoff: 3500 Da, diameter: 22 mm) and dialyzed against deionized water for three days. The dialysate was collected and concentrated. The concentration of RhB in the concentrated dialysate was determined by constructing a standard curve based on the fluorescence response values of RhB at various concentrations. In conjunction with the feeding molar ratio, it was calculated that approximately three RhB molecules are conjugated to each PLL molecule in RhB-PLL.

**Fig. S10.** Rhodamine B (RhB) standard curve

**4. Charge neutralization of RhB-PLL by EDTA confirmed by zeta potential measurement**


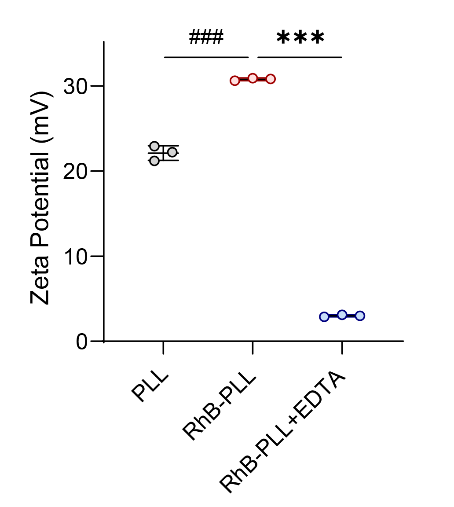


**Fig. S11.** Zeta potential of Rhodamine B-conjugated poly-L-lysine (RhB-PLL) after incubation with Ethylenediaminetetraacetic acid (EDTA). Statistical significance was set at *P* < 0.05. ^###^*P* < 0.001 compared with the PLL group, ^***^*P* < 0.001 compared with the RhB-PLL group, n = 3.

**5. Table S1 lists the name of each group, the treatment method, the dose, and the n value.**

**Table S1.** Experimental grouping and treatment protocol in animal experiments.

| Name | Treatment method & dose | Number of mice | Methods |
| --- | --- | --- | --- |
| Fig. 2 | | | |
| control | i.p. saline | n = 6 | i.v. RhB or RhB-PLL 25mg/kg. For in vivo imaging, lung tissues were collected. |
| model | *PA* 14 infection;  i.p. saline | n = 6 |  |
|  | *PA* 14 infection;  i.p. Ceftazidime 400 mg/kg and avibactam 100 mg/kg | n = 6 |  |
| Fig. 3,4 | | | |
| control | i.p. saline | n = 5 | i.v. RhB-PLL 25mg/kg, for in vivo imaging, lung tissue was harvested, and blood was used for metabolomics analysis. |
| model (8 h, 12 h, 16 h, 24 h) | *PA* 14 infection for 8 h, 12 h, 16 h, 24 h;  i.p. saline | n = 5 |  |
| ceftazidime + avibactam | *PA* 14 infection;  i.p. ceftazidime 400 mg/kg and avibactam 100 mg/kg | n = 5 |  |
| Fig. 6,7 | | | |
| control | i.p. saline | n = 6 | i.v. RhB-PLL 25mg/kg, for in vivo imaging. lung tissue was harvested, and blood was used for metabolomics analysis (n = 5). |
| model (8 h, 12 h, 16 h, 24 h) | *PA* 14 infection for 8 h, 12 h, 16 h, 24 h;  i.p. saline | n = 6 |  |
| ceftazidime + avibactam | *PA* 14 infection;  i.p. ceftazidime 400 mg/kg and avibactam 100 mg/kg | n = 6 |  |
| FZJD | *PA* 14 infection;  Oral FZJD decoction, 22 g/kg | n = 6 |  |
| XCH | *PA* 14 infection;  Oral XCH decoction, 9 g/kg | n = 6 |  |
| SR | *PA* 14 infection;  Oral SR decoction, 7 g/kg | n = 6 |  |
| MXSG | *PA* 14 infection;  Oral MXSG decoction, 7 g/kg | n = 6 |  |

RhB-PLL: Rhodamine B-conjugated poly-L-lysine; PA 14: *P. aeruginosa* 14; FZJD: Fuzheng Jiedu decoction; XCH: Xiaochaihu decoction; SR: Sanren decoction; MXSG: Maxingshigan decoction; i.p.: intraperitoneal.

**6. Stability of RhB-PLL nanoparticles in PBS and serum-containing PBS**

Materials: The fetal bovine serum were purchased from Beijing TransGen Biotech Co., Ltd. (Beijing, China).

RhB-PLL nanoparticles were incubated in PBS or 10% serum-containing PBS, and their average particle size and zeta potential were measured at multiple time points over a 48-hour period.


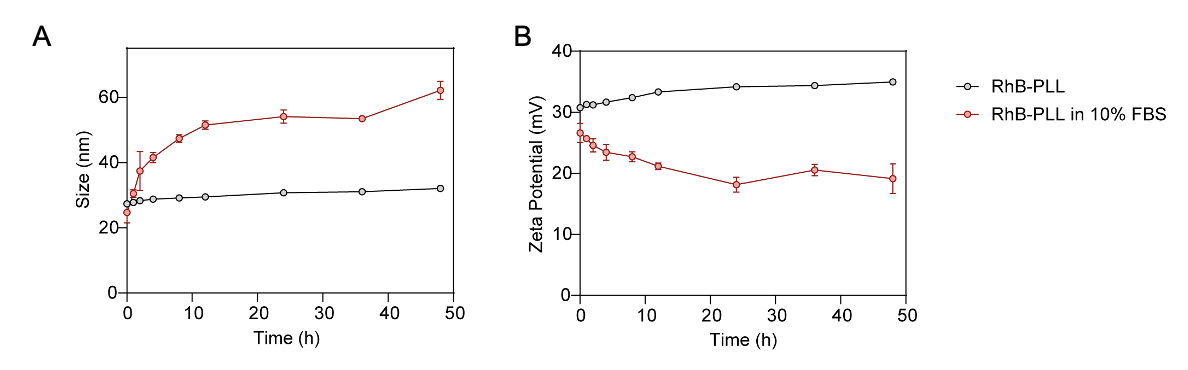


**Fig. S12.** Stability of Rhodamine B-conjugated poly-L-lysine (RhB-PLL) nanoparticles in serum-containing phosphate-buffered saline (PBS)

FBS: Fetal Bovine Serum.

**7. Establish of acute pulmonary inflammation mice model**

The survival rate of mice in the model group was only 20% 24 hours after modeling. The mice in the CAT group were in better condition, and the survival rate reached 80% after 24 hours.


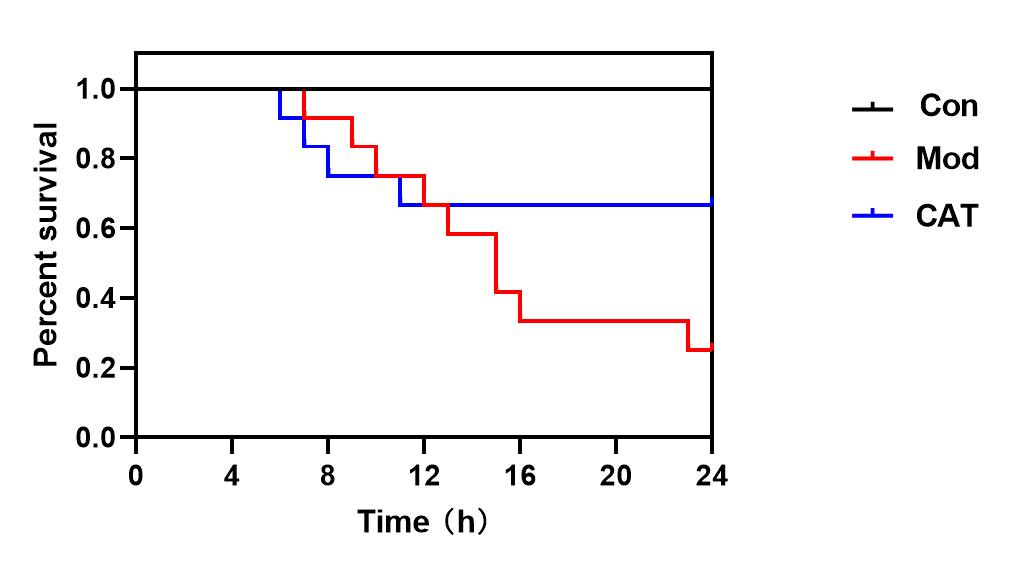


**Fig. S13.** 24 hours survival curves of the Acute Lung Injury (ALI) Mod group and the Ceftazidime plus avibactam (CAT) group, n = 12. Con: Control; Mod: Model; CAT: Ceftazidime plus avibactam.

**8.** **Evans blue experiment**

The Evans blue method was conducted in accordance with the literature. Twenty-four hours after modeling, animals in each group were injected with Evans blue solution (30 mg/kg) via the tail vein. After 40 minutes, they were all sacrificed uniformly. The heart and lung tissues were exposed by thoracotomy, and cardiac perfusion was performed with PBS until the effluent was clear and bloody. Take out the lung tissue and add 10% formamide (dosage of formamide: The lung tissue (1 mL/100 mg) was extracted in a 60°C water bath for 18 hours, centrifuged at 10,000 rpm for 10 minutes, and the supernatant was collected. The absorbance values at 620 nm and 740 nm were determined using an enzyme-labeled instrument (TECAN, Austrian). Establish the standard curve of Evans blue content - absorbance. Calculate the Evans blue content according to the following formula:

*Evans Blue = A620nm-(1.426×A720nm+0.03)*


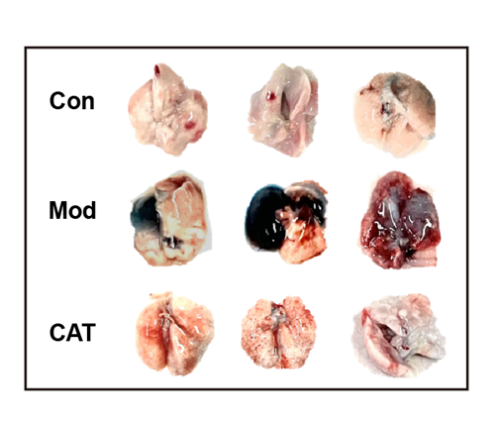


**Fig. S14.** The Evans blue method was used to detect the changes of pulmonary vascular. Con: Control; Mod: Model; CAT: Ceftazidime plus avibactam.

**
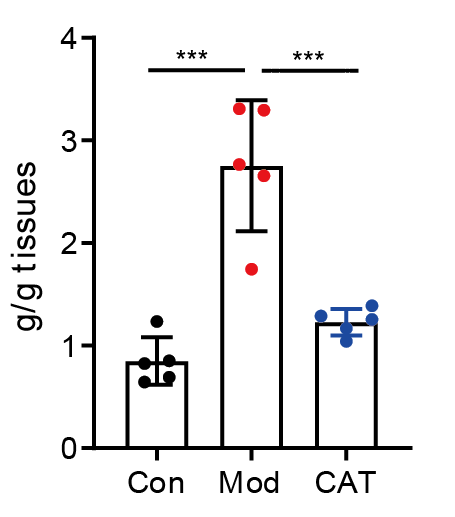
**

**Fig. S15.** The statistics of changes in pulmonary vascular permeability detected by the Evans blue method in acute lung injury. Data presented as mean ± standard deviation (SD). Statistical significance was set at *P* < 0.05. ^***^*P* < 0.001, n = 5. Con: Control; Mod: Model; CAT: Ceftazidime plus avibactam.

**9. RhB-PLL accumulation in pulmonary cells**

Materials: CD68 Monoclonal Antibody (66231-2-Ig) were purchased from Proteintech Wuhan Sanying (Wuhan, China), E cadherin Polyclonal Antibody (bs-10009R) were purchased from Bioss Biotechnology Co., Ltd. (Beijing, China).

Immunofluorescence staining was performed to visualize the accumulation of RhB-PLL nanoparticles in pulmonary macrophages (CD68, green) and epithelial cells (E-cadherin, red) at 16 hours post-administration.


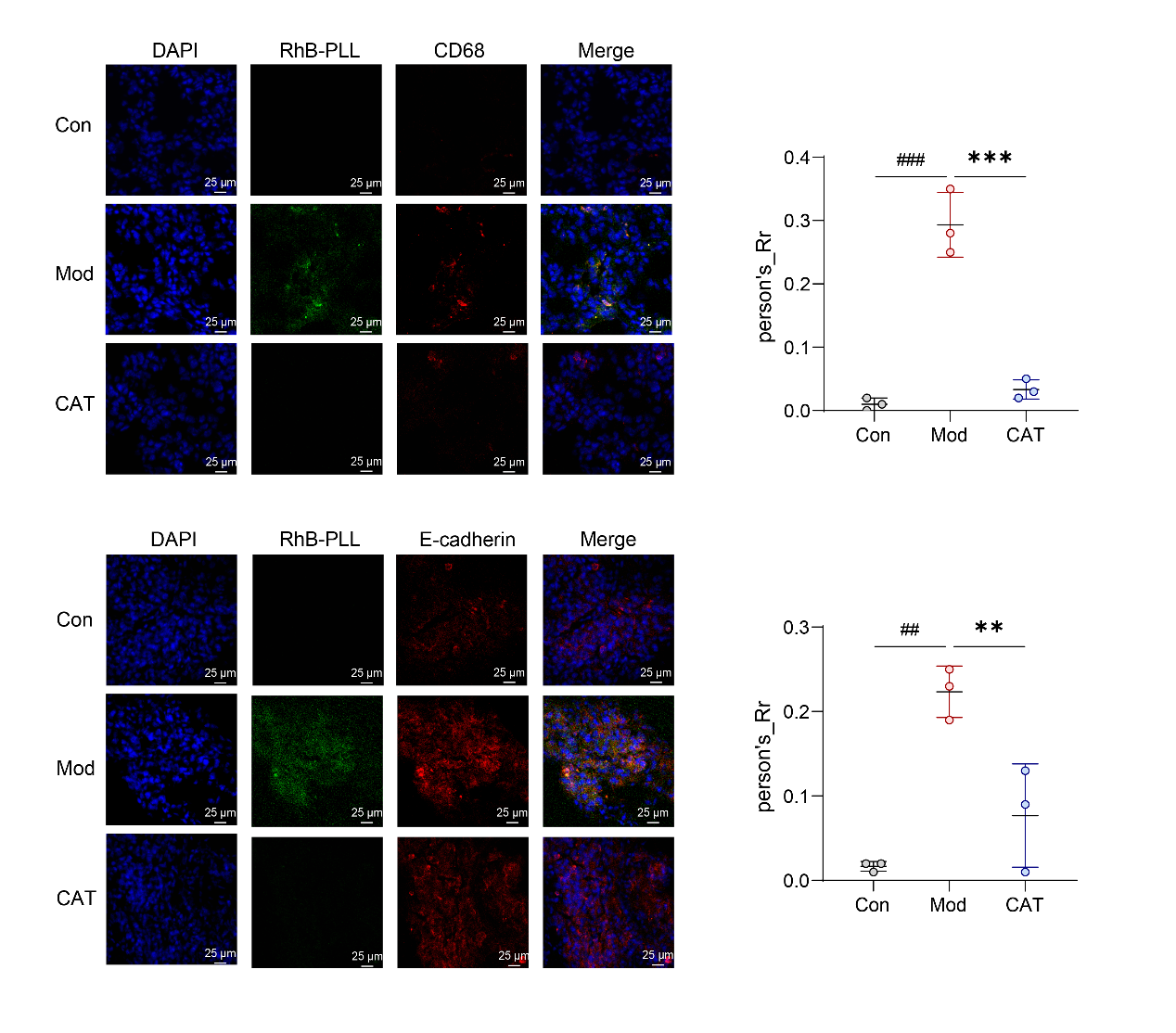


**Fig. S16.** Cellular localization of Rhodamine B-conjugated poly-L-lysine (RhB-PLL) in lung tissue. Statistical significance was set at *P* < 0.05. ^##^*P* < 0.01, ^###^*P* < 0.001 compared with the Con group, ***P* < 0.01, ****P* < 0.001 compared with the Mod group, ns: not significant, n=3. Con: Control; Mod: Model; CAT: Ceftazidime plus avibactam.

**10. Detection of the distribution of RhB-PLL in major organs**

Fluorescence imaging of major organs (heart, liver, spleen, lung, kidney) at multiple time points post-injection of RhB-PLL in healthy mice.


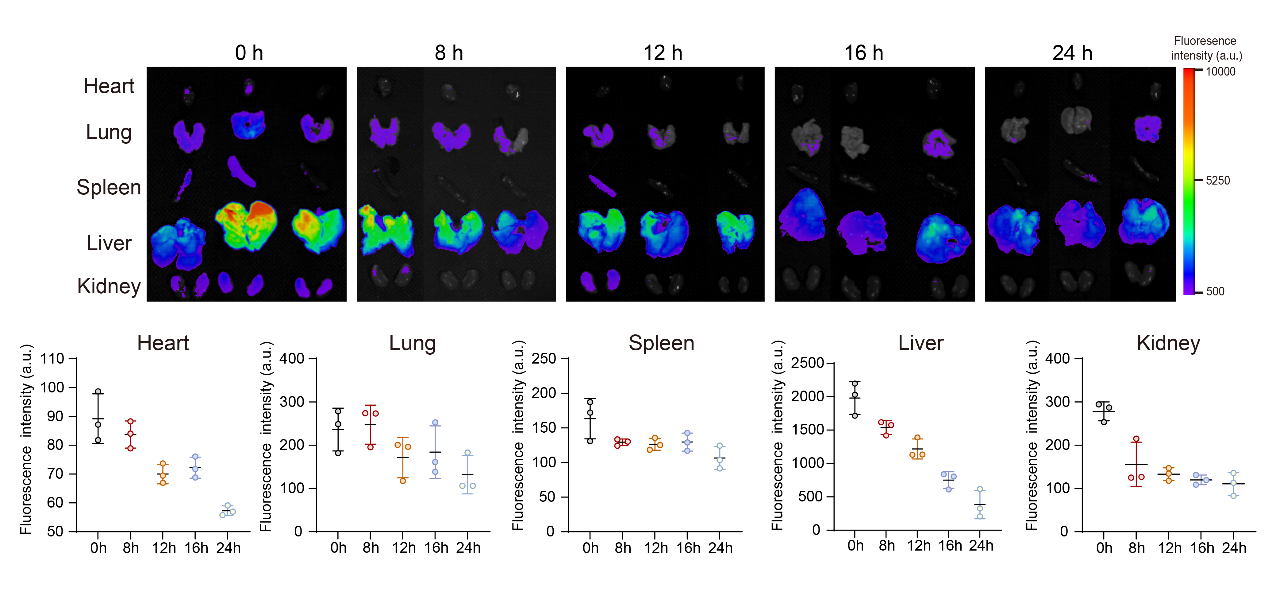


**Fig. S17.** Biodistribution of Rhodamine B-conjugated poly-L-lysine (RhB-PLL) nanoparticles in healthy mice over 24 hours, n=3.

**11. Analysis of mitochondrial targeting of RhB-PLL on cells**

Materials: MitoTracker Deep Red FM(M22426) were purchased from Thermo Fisher Scientific Inc (Grand Island, New York, USA).

To assess the mitochondrial targeting ability of RhB-PLL, RAW264.7 macrophages were stimulated with lipopolysaccharide (LPS, 1 μg/mL) for 12 hours to induce an inflammatory state. Cells were then incubated with RhB-PLL, RhB alone, or RhB-PLL pretreated with 2 mM EDTA (to neutralize surface charge) for 2 hours at 37°C. CAT-treated groups served as positive controls. Following incubation, cells were stained with MitoTracker Green and imaged using confocal laser scanning microscopy. Colocalization analysis was performed to evaluate the extent of RhB-based fluorescence within mitochondria.


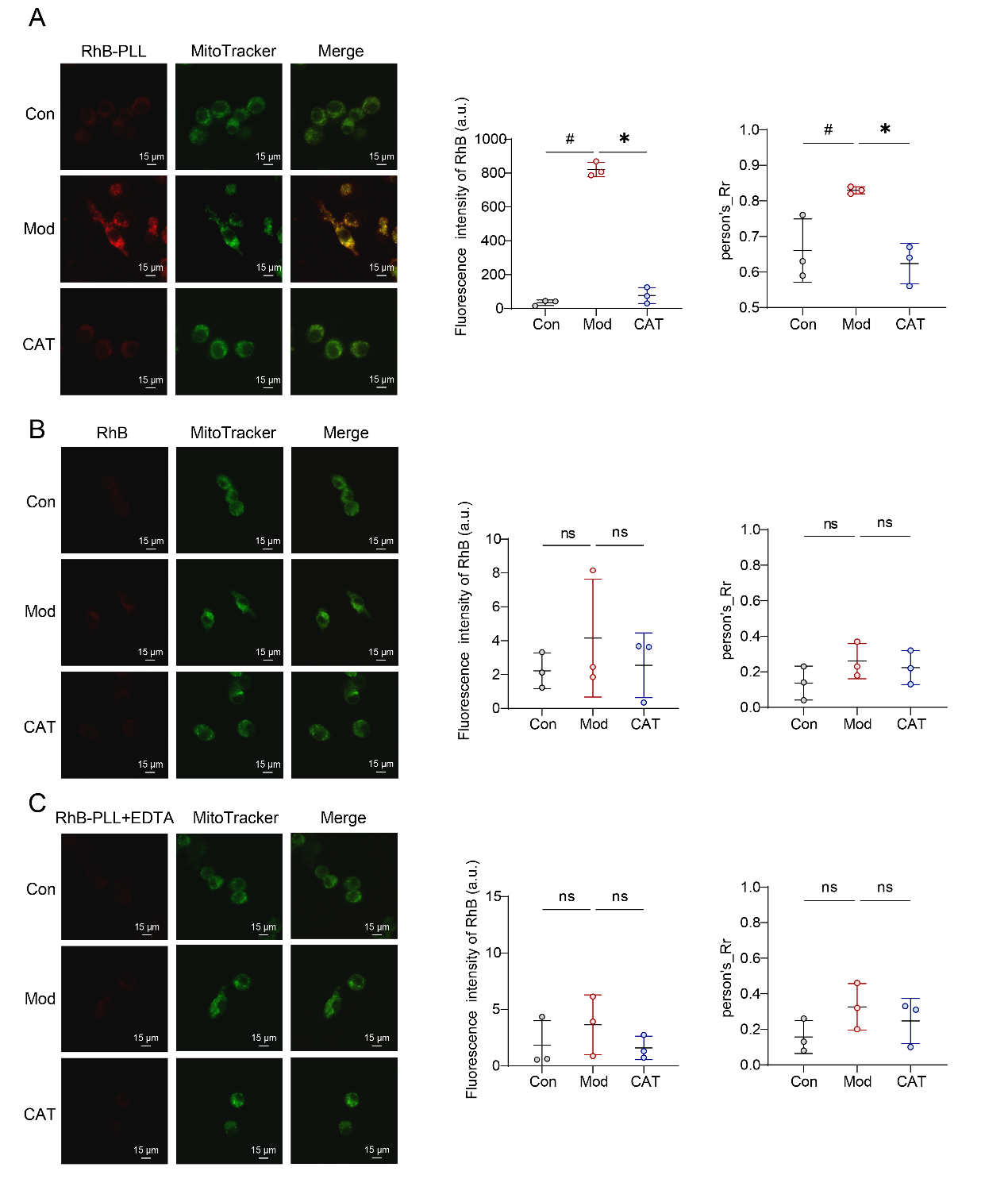


**Fig. S18.** Evaluation of mitochondrial targeting capability of Rhodamine B-conjugated poly-L-lysine (RhB-PLL) in Lipopolysaccharide (LPS)-induced RAW264.7 cells. EDTA: Ethylenediaminetetraacetic acid. Statistical significance was set at *P* < 0.05. ^#^*P* < 0.05 compared with the Con group, **P* < 0.05 compared with the Mod group, ns: not significant, n = 3. Con: Control; Mod: Model; CAT: Ceftazidime plus avibactam.

**12. OPLS-DA component analysis of ALI mice at each time point**

Metabolites were identified and orthogonal partial least squares discriminant analysis (OPLS-DA) was performed with the aid of SIMCA software. The results showed that the intra-group clustering was obvious and the samples in different groups were well dispersed among the groups.

**
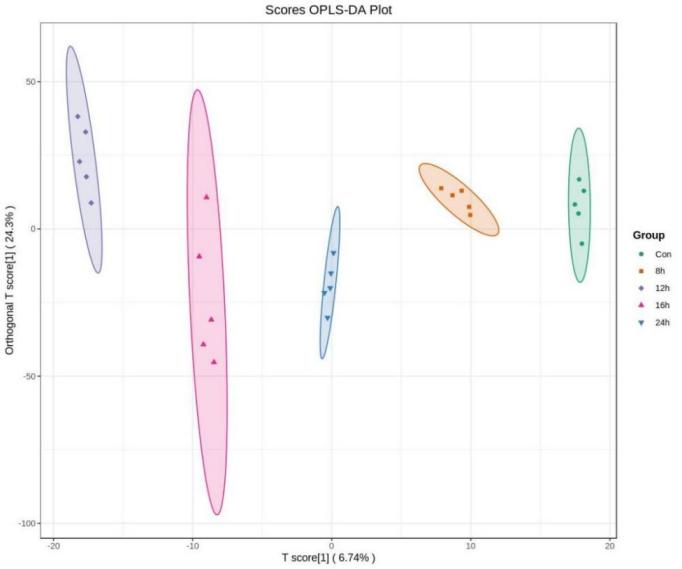
**

**Fig. S19.** Principal component analysis of Orthogonal Partial Least Squares Discriminant Analysis (OPLS-DA) in lung tissue at each time point of acute lung injury (ALI) , n = 5. Con: Control.

**13. PCA cluster analysis of six potential biomarkers**

PCA cluster analysis was performed on the six differentially metabolites at each time point. The results were similar to those of OPLS-DA, and the discrimination among the groups was good, indicating their potential as new biomarkers for the staging of pulmonary inflammation.


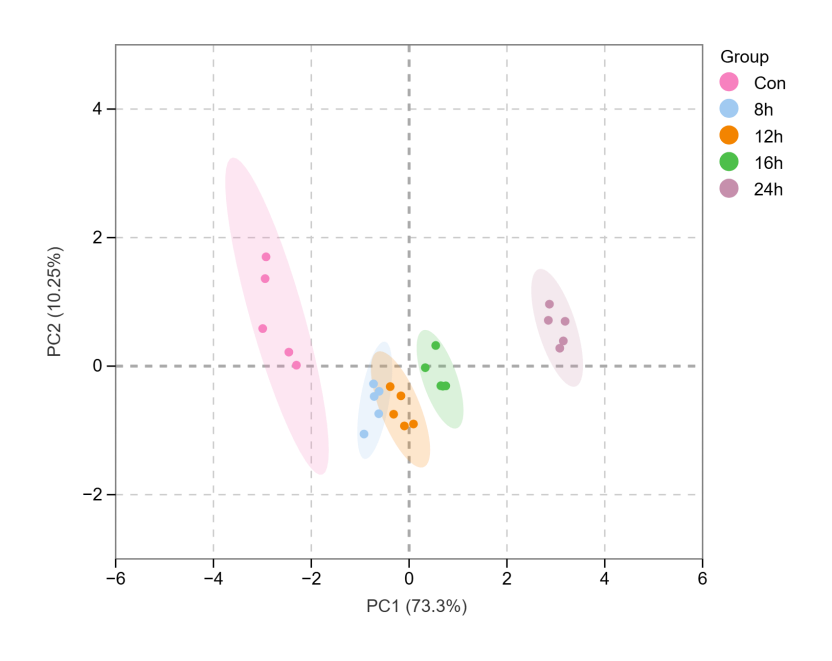


**Fig. S20.** Principal component analysis (PCA) of lung tissue at each time points of acute lung injury (ALI) (n = 5). Con: Control.

**14. Acute toxicity** **evaluation of RhB-PLL**

Male KM mice were randomly divided into two groups (n = 5): the Control group (i.v., saline) and the RhB-PLL group (i.v., RhB-PLL, 25 mg/kg). Following administration, the body weight of the mice was monitored every other day. On day 15, the mice were euthanized, and blood samples were collected for hepatic and renal function analysis using comprehensive test disks for plasma index assays (Dymind, China). Organs including the heart, liver, spleen, lung, and kidney were harvested, weighed, and subjected to hematoxylin and eosin (H&E) staining for pathological evaluation.


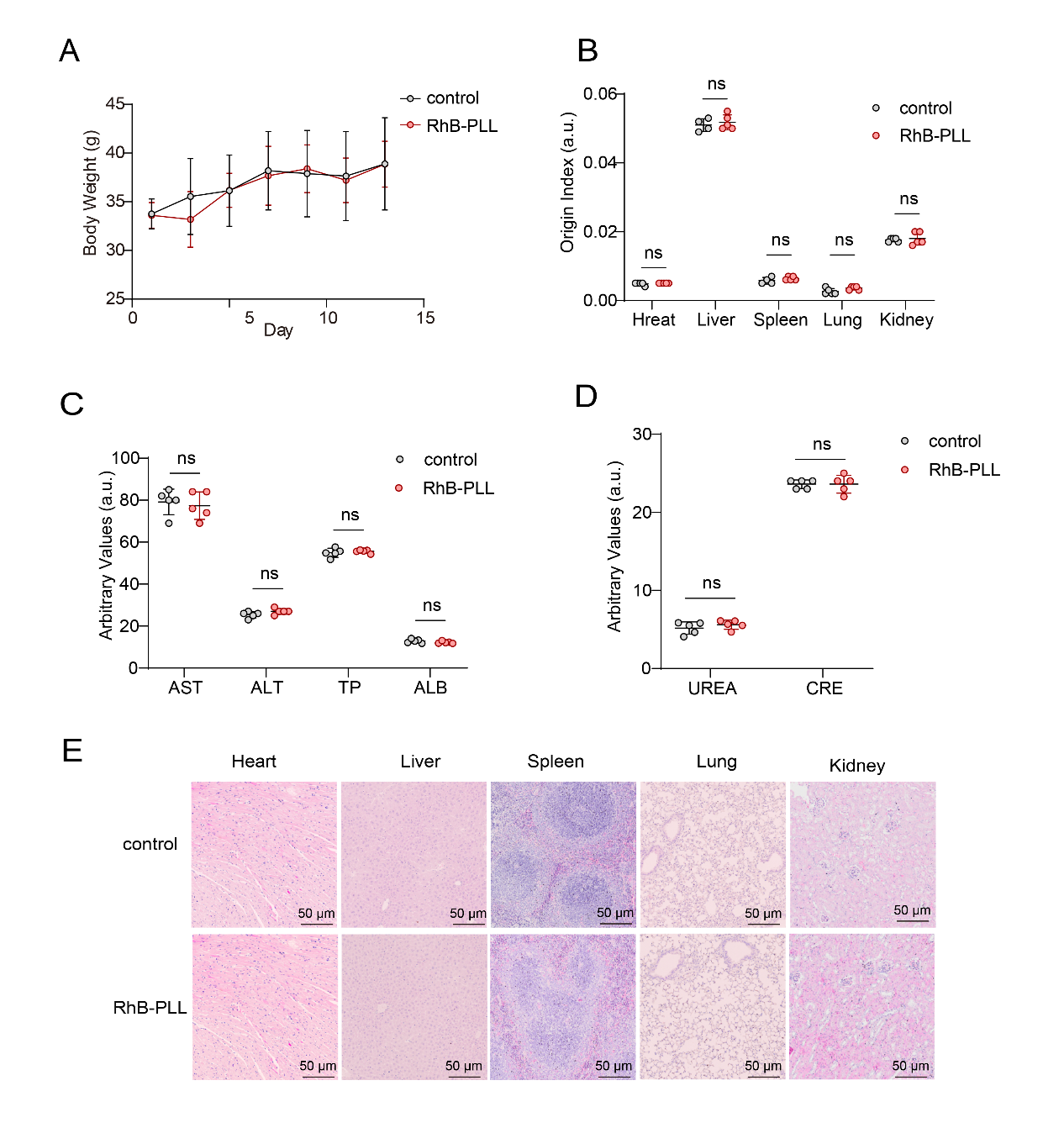


**Fig. S21.** *In-vivo* safety evaluation of Rhodamine B-conjugated poly-L-lysine (RhB-PLL). (A) Weight change over a 14-day period. (B) Organ index. (C) Evaluation of biochemical indicators of liver function including aspartate aminotransferase (AST), alanine aminotransferase (ALT), total protein (TP), and albumin (ALB). (D) Evaluation of biochemical indicators of renal function including UREA and creatinine (CRE). (E) HE staining of major organ tissues. Statistical significance was set at *P* < 0.05, ns: not significant, n = 5.
